# CRISPR interference interrogation of COPD GWAS genes reveals the functional significance of desmoplakin in iPSC-derived alveolar epithelial cells

**DOI:** 10.1126/sciadv.abo6566

**Published:** 2022-07-13

**Authors:** Rhiannon B. Werder, Tao Liu, Kristine M. Abo, Jonathan Lindstrom-Vautrin, Carlos Villacorta-Martin, Jessie Huang, Anne Hinds, Nathan Boyer, Esther Bullitt, Marc Liesa, Edwin K. Silverman, Darrell N. Kotton, Michael H. Cho, Xiaobo Zhou, Andrew A. Wilson

**Affiliations:** ^1^ Center for Regenerative Medicine, Boston University and Boston Medical Center, Boston, MA 02118, USA.; ^2^ Pulmonary Center and Department of Medicine, Boston University School of Medicine, Boston, MA 02118, USA.; ^3^ QIMR Berghofer Medical Research Institute, Herston, QLD 4006, Australia.; ^4^ Channing Division of Network Medicine, Department of Medicine, Brigham and Women’s Hospital, Harvard Medical School, Boston, MA 02115, USA.; ^5^ Department of Physiology and Biophysics, Boston University School of Medicine, Boston, MA 02118, USA.; ^6^ Department of Medicine, Endocrinology, David Geffen School of Medicine at University of California Los Angeles, Los Angeles, CA 90095, USA.; ^7^ Institut de Biologia Molecular De Barcelona (IBMB-CSIC), Barcelona, Catalonia 08028, Spain.

## Abstract

Genome-wide association studies (GWAS) have identified dozens of loci associated with chronic obstructive pulmonary disease (COPD) susceptibility; however, the function of associated genes in the cell type(s) affected in disease remains poorly understood, partly due to a lack of cell models that recapitulate human alveolar biology. Here, we apply CRISPR interference to interrogate the function of nine genes implicated in COPD by GWAS in induced pluripotent stem cell–derived type 2 alveolar epithelial cells (iAT2s). We find that multiple genes implicated by GWAS affect iAT2 function, including differentiation potential, maturation, and/or proliferation. Detailed characterization of the GWAS gene *DSP* demonstrates that it regulates iAT2 cell-cell junctions, proliferation, mitochondrial function, and response to cigarette smoke–induced injury. Our approach thus elucidates the biological function, as well as disease-relevant consequences of dysfunction, of genes implicated in COPD by GWAS in type 2 alveolar epithelial cells.

## Teaser

Interrogation of genes associated with COPD reveals functional consequences in iPSC-derived type 2 alveolar epithelial cells.

## INTRODUCTION

Chronic obstructive pulmonary disease (COPD) is a major cause of morbidity and mortality and the third leading cause of death worldwide (*1*). The disease encompasses both chronic bronchitis and emphysema resulting from injury to the two major lung compartments, the airways and the alveoli. COPD results from a combination of environmental exposures and genetic susceptibility. While the primary environmental triggers, including inhaled tobacco smoke and indoor pollution from burning coal and/or biomass, have long been established, much about disease susceptibility remains unknown. Most long-term smokers do not develop severe COPD (*2*), and susceptibility is a heritable trait (*3*), underscoring the contribution of genetic factors in disease pathogenesis. Furthermore, studies have determined that COPD development may commence early in life, as approximately half of COPD patients identified among three longitudinal cohort studies exhibited an abnormally low baseline forced expiratory volume in the first second (FEV1) in early adulthood (*4*). In some instances, COPD can occur in the absence of known environmental exposures, further suggesting a genetic cause (*5*, *6*). Substantial overlap exists among risk loci for COPD and loci associated with population-based lung function (*7*–*11*). Moreover, COPD-implicated genetic loci are enriched for regions involved in lung development (*7*, *9*, *12*), suggesting a potential role in disease pathogenesis for genes central to lung development.

Given the mounting evidence that genetic factors underlie COPD susceptibility, there is an unmet need to investigate the biology of potential genetic contributors in the lung to COPD, including genome-wide association studies (GWAS) genes. GWAS identify genomic regions containing risk variants for a trait of interest, but additional analyses are required to identify the functional genes within GWAS loci. A significant barrier to performing such studies has been a lack of human models that faithfully recapitulate the biology of cell types linked to COPD pathogenesis, such as the type 2 alveolar epithelial cell (AT2) (*13*, *14*). Genetic loci implicated in COPD by GWAS are enriched in AT2s (*11*, *15*), suggesting them as a rational target for functional genetic studies to delineate cellular events associated with disease inception or progression. Differences between murine and human lung structure (*16*), genetics (*17*), and manifestation of disease (*18*, *19*) underscore the need for human cellular systems to model AT2 dysfunction contributing to disease pathogenesis. However, investigations of primary human AT2s have been limited as they are difficult to access, maintain in culture, and manipulate genetically. The discovery of induced pluripotent stem cells (iPSCs) and the application of their differentiated progeny have now begun to address this challenge. Directed differentiation of iPSCs produces an inexhaustible supply of disease-relevant cell types for the study of GWAS signals (*20*). To model respiratory diseases in vitro (*21*–*23*), we and others have developed protocols to differentiate and mature human AT2s derived from iPSCs (iAT2s) that transcriptomically overlap with adult human primary AT2s, execute critical cell type–specific functions, and recapitulate disease-specific phenotypes of the human subject of origin (*22*). Here, we apply CRISPR interference (CRISPRi) to determine the functional consequences of knocking down expression of GWAS genes of interest in iPSC-derived lung progenitor cells and iAT2s. Using this approach, we identify multiple genes that alter expression of the iAT2 transcriptomic program and find that desmoplakin (*DSP*) regulates iAT2 cell-cell junctions, mitochondrial function, proliferation, and response to injury.

## RESULTS

### CRISPRi-mediated knockdown of COPD GWAS genes alters the iAT2 transcriptional program

**Fig. 1.**
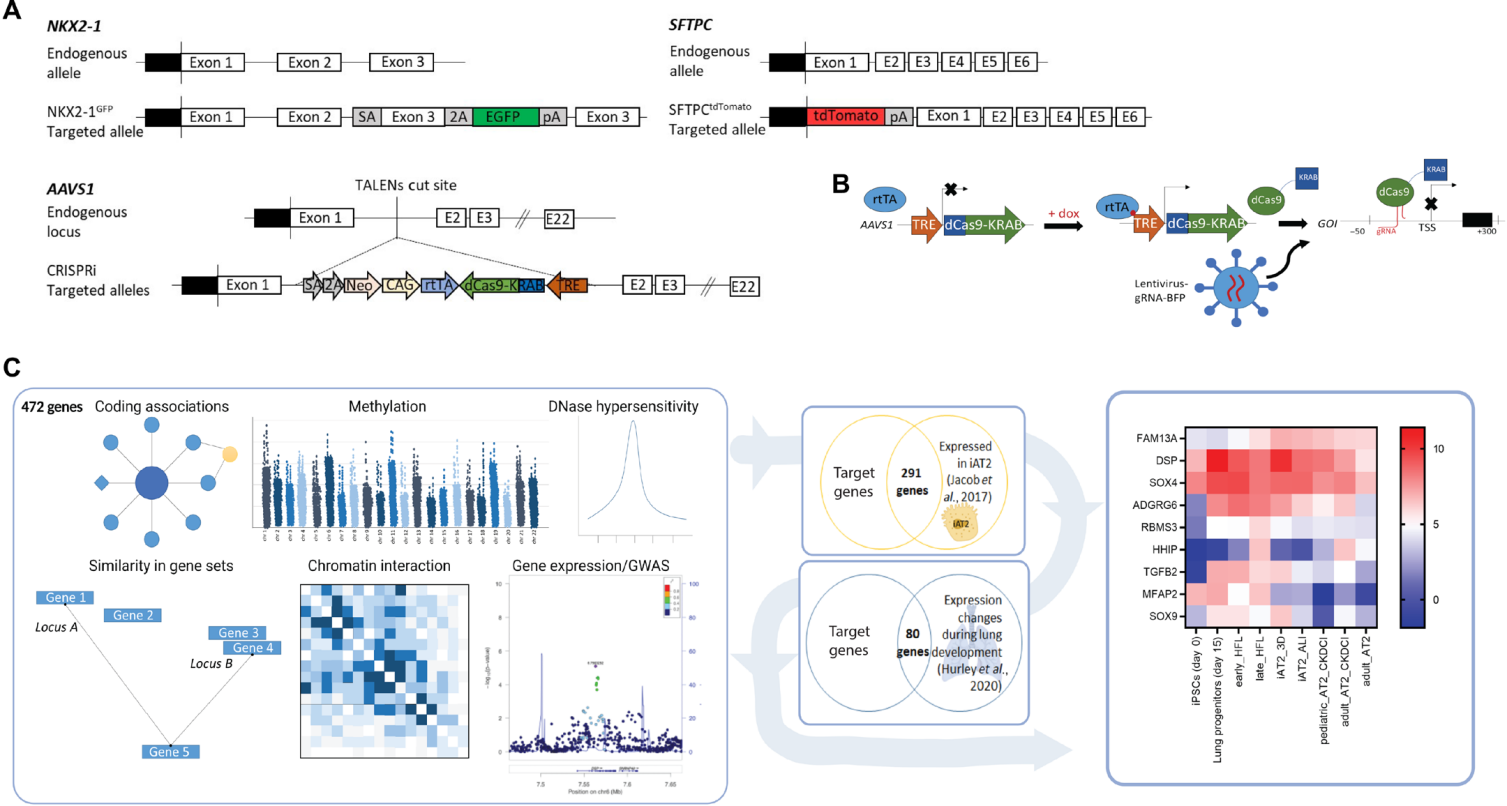
Overview of CRISPRi platform to interrogate COPD GWAS genes. (**A**) Gene editing strategy to generate inducible CRISPRi iPSC/ESC lines with an NKX2-1–GFP and/or SFTPC-tdTomato reporter. (**B**) Schematic representation of dox-inducible dCas9-KRAB expression and lentiviral-delivered gRNA targeted to the transcriptional start site (TSS) of genes of interest (GOI) to mediate knockdown. (**C**) Effector genes at COPD genome-wide significant loci were identified using gene expression, methylation status, coding associations, deoxyribonuclease (DNase) hypersensitivity, chromatin interactions, and/or similarity in gene sets, as previously described (*11*). Genes were further filtered on the basis of expression during lung-directed differentiation of iPSCs, expression in differentiated iAT2s, and expression changes during human lung development. Expression levels of the final selected genes, FAM13A, DSP, TGFB2, MFAP2, RBMS3, SOX4, SOX9, HHIP, and ADGRG6, are shown in iPSCs, iPSC-lung progenitors, early (16 to 17.5 weeks of gestation) and late (20 to 21 weeks of gestation) human fetal lung (HFL), iAT2s grown as 3D alveolospheres or at air-liquid interface (ALI), primary pediatric (13-month-old male donor) and adult (32-year-old male donor) AT2s grown in CK + DCI with MRC-5 cells, and freshly isolated primary adult AT2s (*26*).

**Fig. 2.**
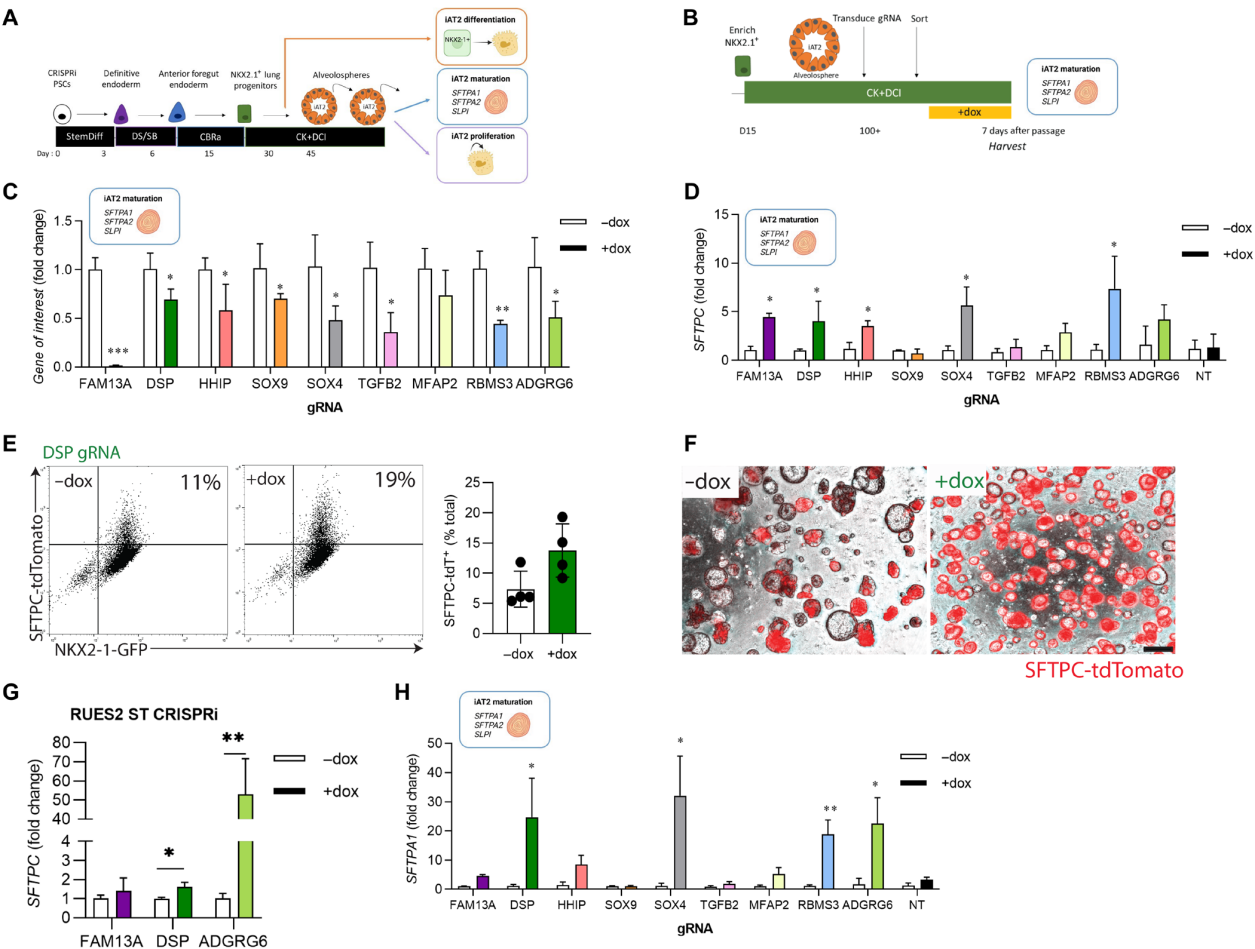
COPD GWAS candidate genes can be interrogated by CRISPRi. (**A**) Schematic representation of proposed experiments to interrogate the function of genes of interest in iAT2s. iPSCs undergo directed differentiation to generate NKX2-1^+^ lung progenitors, which are then sorted and replated in distal medium (CK + DCI). iAT2s are harvested at either early or later time points to assess the effect of target genes on specific end points as indicated. (**B**) CRISPRi-iPSC–derived iAT2s were transduced with lentiviral-gRNA, and transduced cells were sorted 7 days later based on lenti-BFP expression. After recovery and expansion, iAT2s were treated with dox for 7 to 21 days to initiate CRISPRi-knockdown and harvested 7 days after passage. (**C**) Expression of each gene of interest following dox treatment as assessed by qRT-PCR, relative to control (−dox) iAT2s. (**D**) Expression of SFTPC following knockdown of each gene of interest as assessed by qRT-PCR relative to control (−dox) iAT2s. (**E**) Representative flow cytometry plots for iAT2s transduced with DSP gRNA treated without (−dox) or with (+dox) doxycycline showing NKX2-1–GFP and SFTPC-tdTomato expression. Cells were gated on nonfragmented single cells. (**F**) Representative live-cell imaging of iAT2s transduced with DSP gRNA treated without (−dox) or with (+dox) doxycycline (bright-field/SFTPC-tdTomato overlay; scale bar, 100 μm). (**G**) SFTPC expression following knockdown in RUES2 ST CRISPRi iAT2s. (**H**) Expression of SFTPA1 expression following knockdown of each gene of interest, assessed by qRT-PCR relative to control (−dox) iAT2s. NT, non-targeting gRNA. *n* = 3 experimental replicates of independent wells of a differentiation; error bars represent SD. Statistical significance was determined by unpaired, two-tailed Student’s *t* test; *P < 0.05 and **P < 0.005.

To interrogate gene function, we first engineered an inducible CRISPRi system that would accomplish knockdown in all cells with temporal control. We targeted the *AAVS1* locus of previously established embryonic stem cell (ESC) and iPSC lines containing lung lineage fluorochrome reporters (NKX2-1^GFP^ and/or SFTPC^tdTomato^) (*22*) with a construct encoding dCas9-KRAB under the control of a TRE promoter along with a CAG-driven rtTA (Fig. 1A) (*24*). Successful integration of the CRISPRi construct in individual iPSC/ESC clones was confirmed by polymerase chain reaction (PCR), Sanger sequencing, and Southern blot (fig. S1, A and B), and selected clones were karyotypically normal (fig. S1C). Exposure of CRISPRi iPSCs to doxycycline (dox)–induced dCas9 protein expression (fig. S1D) and dCas9 remained inducible throughout lung-directed differentiation (fig. S1E). To target dCas9-KRAB to genes of interest, we used a lentiviral vector that can coexpress four guide RNA (gRNAs) from individual promoters, together with an EBFP2 reporter to identify transduced cells [adapted from (*25*)] (Fig. 1B). This approach generated efficient and long-term knockdown of *SFTPC* in iAT2s (fig. S1F), meaning that CRISPRi-mediated knockdown could be successfully used in the directed differentiation of lung progenitors and iAT2s. To select genes for further study, we started with a list of 472 previously identified genes associated with COPD and lung function based on genome-wide significance in combination with gene expression, methylation status, coding associations, deoxyribonuclease (DNase) hypersensitivity, chromatin interactions, and/or similarity in gene sets (Fig. 1C) (*11*). We then selected genes expressed in both primary AT2s and differentiated iAT2s with expression levels that changed over the course of human lung development (*26*). Informed by known biology of remaining genes, we ultimately selected nine genes for further evaluation (Fig. 1C). We then applied established protocols to derive iPSC-NKX2-1^+^ lung epithelial progenitors and their differentiated SFTPC^+^ progeny, iAT2s (*22*, *27*, *28*). To explore the contribution of each target gene to iAT2 phenotypes (Fig. 2A), differentiated iAT2s were transduced with lentiviral-gRNA targeting the transcriptional start site (TSS) of each gene of interest and sorted to generate pure populations of gRNA-expressing cells (Fig. 2B). Knockdown was initiated by the addition of dox for 7 or more days, and cells were collected at 7 days after passage. gRNAs designed to target the TSS of genes of interest induced statistically significant knockdown of eight of nine genes in iAT2s (Fig. 2C), providing a solid cellular platform to evaluate effects of various COPD GWAS genes on iAT2 characteristic function.

Expression of *SFTPC*, a canonical AT2 marker gene, was significantly increased by knockdown of *FAM13A*, *DSP*, *HHIP*, *SOX4*, and *RBMS3* (Fig. 2D), while knockdown of other genes of interest or expression of a nontargeting (NT)–gRNA control had no effect. The observed increase in *SFTPC* mRNA was reflected by both increased intensity of expression and proportion of cells expressing the SFTPC^tdTomato^ reporter, exemplified by knockdown of *DSP*, which increased the proportion of tdTomato^+^ iAT2s (Fig. 2, E and F). Reproducibility of the effects of knockdown on *SFTPC* expression was confirmed for a subset of these genes in a second genetically distinct PSC line, RUES2 ST CRISPRi (Fig. 2G and fig. S1G). To further elucidate the effects of gene knockdown on the iAT2 program, we next applied a gene set we have previously identified to be up-regulated during AT2 maturation (*21*), including *SFTPA1*, *SFTPA2*, and *SLPI*. We found that knockdown of *FAM13A*, *DSP*, *HHIP*, *SOX4*, *RBMS3*, and *ADGRG6* significantly increased expression of at least two of three maturation genes (Fig. 2H and fig. S1, H and I), suggesting an augmented AT2 maturation program beyond simply elevated expression of *SFTPC*. To confirm whether the effects of knockdown similarly altered gene expression earlier in directed differentiation, we transduced differentiating iPSCs at the anterior foregut endoderm stage with lentiviral-gRNA constructs and sorted transduced [blue fluorescent protein (BFP)^+^] or nontransduced (BFP^−^) NKX2-1^+^ lung progenitors. Sorted cells were replated in iAT2 differentiation medium containing dox for 2 weeks (fig. S1J). Intriguingly, while knockdown of *DSP* again increased *SFTPC* expression at this stage, knockdown of other genes (*FAM13A*, *HHIP*, *SOX4*, and *RBMS3*) that had done so in established iAT2s did not, suggesting a developmental stage–specific effect of these genes (fig. S1, K and L). Together, these data suggest that multiple COPD-associated GWAS genes, including *FAM13A*, *DSP*, *HHIP*, *SOX4*, *RBMS3*, and *ADGRG6*, influence expression of genes central to AT2 maturation and function.

### CRISPRi-mediated knockdown of COPD GWAS genes alters iAT2 proliferation

**Fig. 3.**
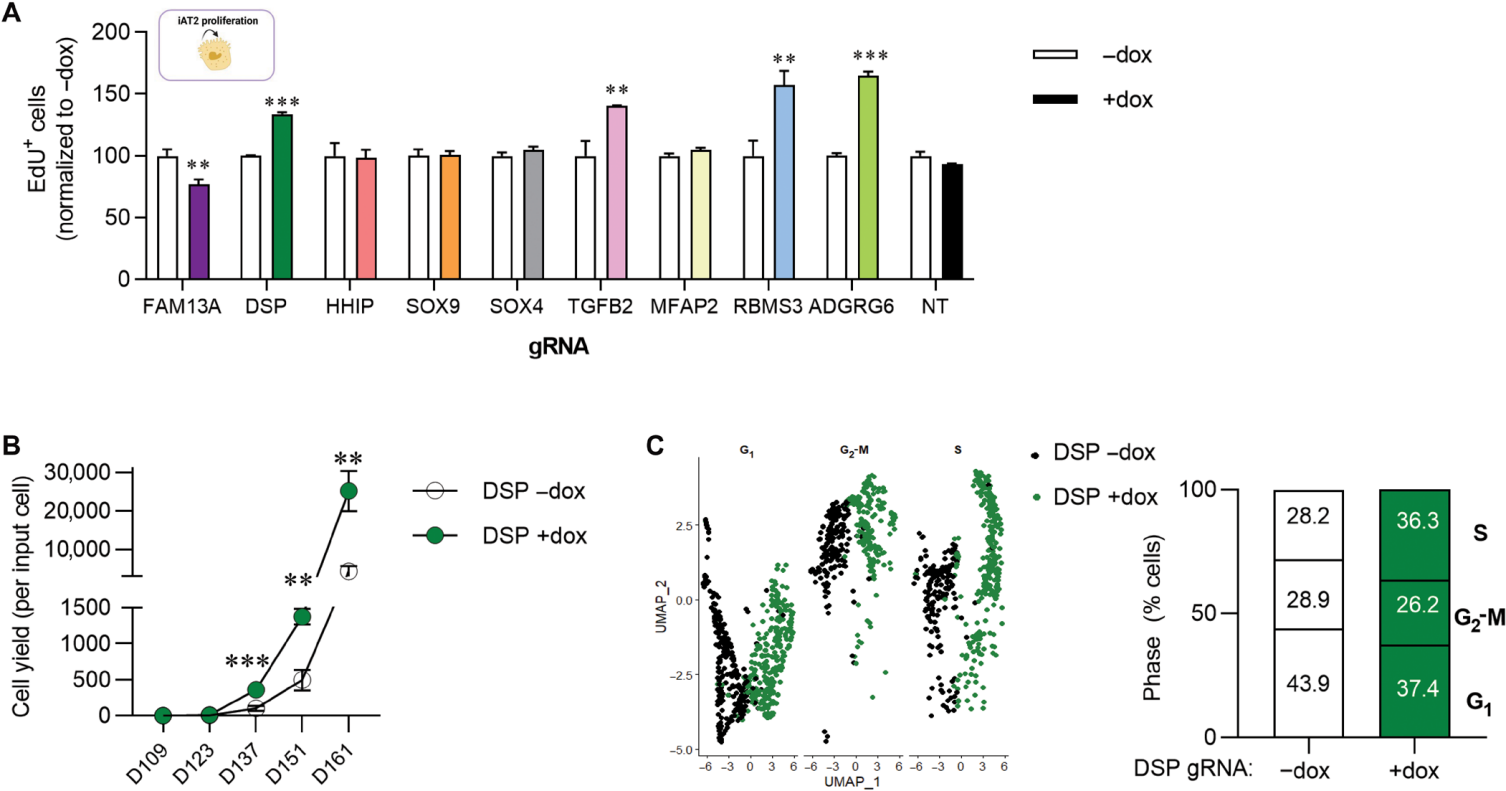
CRISPRi-knockdown of genes of interest modulates iAT2 proliferation. (**A**) CRISPRi-iAT2s were transduced with lentiviral-gRNA as described in Fig. 2. Six days after passage, cells were treated with EdU for 24 hours. EdU incorporation was measured by flow cytometry, and the data were normalized to the control (−dox) iAT2s. (**B**) Graph showing yield per input iAT2 transduced with DSP gRNA and treated without (−dox) or with (+dox) doxycycline across five passages. (**C**) Single-cell RNA sequencing (scRNA-seq) showing cell cycle phase using UMAP of iAT2s transduced with DSP gRNA and treated without (−dox, black) or with (+dox, green) doxycycline. *n* = 3 experimental replicates of independent wells of a differentiation; error bars represent SD. Statistical significance was determined by unpaired, two-tailed Student’s *t* test; **P < 0.005, and ***P < 0.001.

The ability to proliferate is essential to AT2 function in their known role as facultative progenitors of the distal epithelium (*29*). To assess the effect of genes of interest on iAT2 proliferation, we next measured 5-ethynyl-2′-deoxyuridine (EdU) incorporation by flow cytometry. We found that knockdown of *FAM13A* significantly slowed proliferation of iAT2s (Fig. 3A and fig. S2A). In contrast, knockdown of *DSP*, *TGFB2*, *RBMS3*, and *ADGRG6* increased proliferation (Fig. 3A), while for other genes there was no effect (*HHIP*, *SOX9*, *SOX4*, *MFAP2*, and NT control). Together, these data suggest that knockdown of a subset of COPD-associated genes alters iAT2 proliferation. We further evaluated the durability of this effect for *DSP* knockdown (*DSP*-kd) by assessing iAT2 cell yield over multiple passages and found that down-regulation of *DSP* expression resulted in a significantly greater number of cells per input cell (Fig. 3B). To determine the molecular mechanism by which DSP regulates the proliferation, maturation, and differentiation of iAT2s, we performed single-cell RNA sequencing (scRNA-seq) of iAT2s in the presence (+dox) or absence (−dox) of *DSP*-kd. This scRNA-seq analysis showed that a greater proportion of dox-treated iAT2s were in S phase at the time of harvest (Fig. 3C and fig. S2B), consistent with increased EdU incorporation in iAT2 cells with *DSP*-kd (Fig. 2A).

### DSP expression modulates iAT2 transcriptomic program

**Fig. 4.**
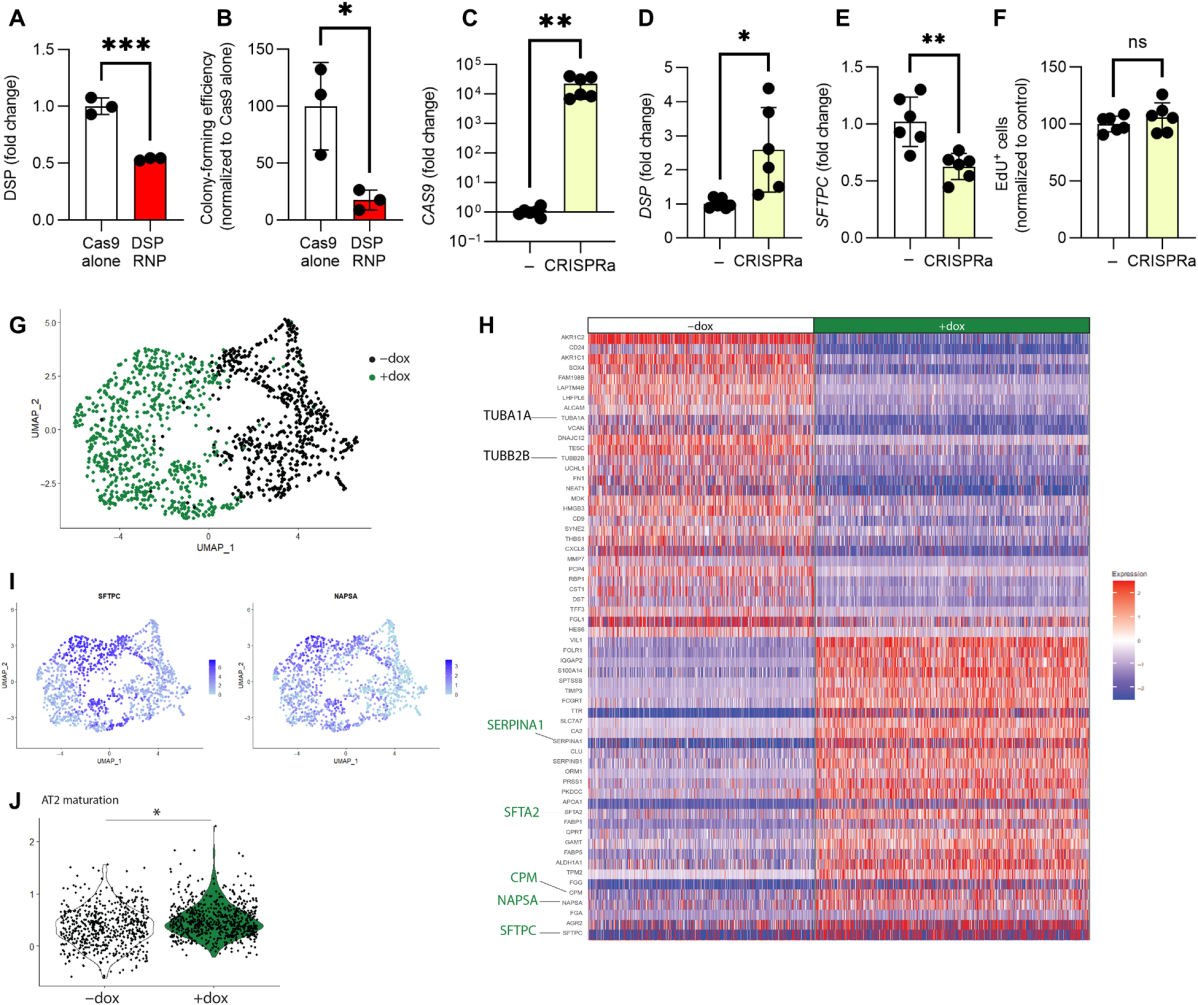
DSP expression modulates iAT2 transcriptome. (**A**) iAT2s were nucleofected with Cas9-gRNA RNP with gRNA targeting exon 1 of DSP. DSP expression was measured by qRT-PCR 48 hours after nucleofection. (**B**) Colony-forming efficiency was determined 2 weeks after nucleofection with RNP. *n* = 3 experimental replicates of independent wells of a differentiation. (**C**) iAT2s were transduced with lentiviral-gRNA targeting the TSS of DSP and sorted to obtain a pure population. iAT2s were then transduced with CRISPRa lentivirus (dCAS9-VP64). Cas9, (**D**) DSP, and (**E**) SFTPC expression were measured by qRT-PCR. (**F**) EdU incorporation was measured by flow cytometry, and the data were normalized to the control (-CRISPRa) iAT2s. *n* = 6 experimental replicates of independent wells of a differentiation. (**G**) UMAP of scRNA-seq, showing original identity (−dox = black, +dox = green). (**H**) Heatmap of scRNA-seq showing top 40 differentially expressed genes between –dox and +dox (green). (**I**) UMAP showing SFTPC and NAPSA expression. (**J**) Module score of AT2 maturation gene set (*21*). All error bars represent SD. Statistical significance was determined by unpaired, two-tailed Student’s *t* test; *P < 0.05, **P < 0.005, and ***P < 0.001.

Our nine-gene CRISPRi screen demonstrated that *DSP* affected all three AT2-associated phenotypes tested (differentiation, maturation, and proliferation). Although DSP is expressed in multiple lung epithelial subtypes, its role in human AT2s has not been studied. Furthermore, the lead COPD GWAS single-nucleotide polymorphism (SNP) at the *DSP* locus has also been associated by GWAS with risk for pulmonary fibrosis (*7*), another disease involving AT2 injury (but with an opposite direction of effect). Recently published scRNA-seq datasets profiling either COPD or pulmonary fibrosis lungs confirmed divergent DSP expression in AT2s in these two disease states relative to controls (fig. S3A) (*30*, *31*). We thus chose to leverage our platform to further investigate the function of DSP in iAT2s. We sought to confirm our knockdown findings by using CRISPR to knock out *DSP* in established iAT2s (Fig. 4A); however, we found that iAT2s did not form alveolospheres or proliferate in the total absence of *DSP* (Fig. 4B), precluding further evaluation using this approach. To provide further context, we next overexpressed *DSP* in iAT2s using CRISPR activation (CRISPRa). We transduced iAT2s with lentiviral-gRNA targeting the *DSP* TSS followed by delivery of lentiviral-CRISPRa (*32*). This approach induced high-level expression of dCas9 (Fig. 4C), and significantly increased *DSP* expression (Fig. 4D) with an associated decrease in *SFTPC* expression (Fig. 4E), but no change in proliferation of iAT2s (Fig. 4F).

To further understand how loss of *DSP* might affect iAT2 function, we further explored our scRNA-seq data of CRISPRi iAT2s transduced with *DSP* lenti-gRNA and treated with dox versus control vehicle. Uniform Manifold Approximation and Projection (UMAP) visualization and Louvain clustering revealed two major clusters, separated by experimental condition, i.e., control (−dox) and *DSP*-kd (+dox) (Fig. 4G and fig. S3B). We identified differentially expressed genes between control (−dox) and *DSP*-kd (+dox) iAT2s and found that many canonical AT2 genes were among the most up-regulated genes in the *DSP*-kd condition (*SFTA2*, *CPM*, *NAPSA*, *SFTPC*, and *SERPINA1)* (Fig. 4, H and I). Moreover, we applied a module score for an AT2 maturation gene set (*21*) and found that this was significantly increased in the *DSP*-kd iAT2s (Fig. 4J). These changes in iAT2 program were not correlated with changes in *NKX2-1* expression (fig. S3C). Together, these data support our previous quantitative reverse transcription PCR (qRT-PCR) results (Fig. 2) and demonstrate that modulating *DSP* expression alters the iAT2 transcriptomic program.

### DSP-kd leads to structural reorganization of iAT2s

**Fig. 5.**
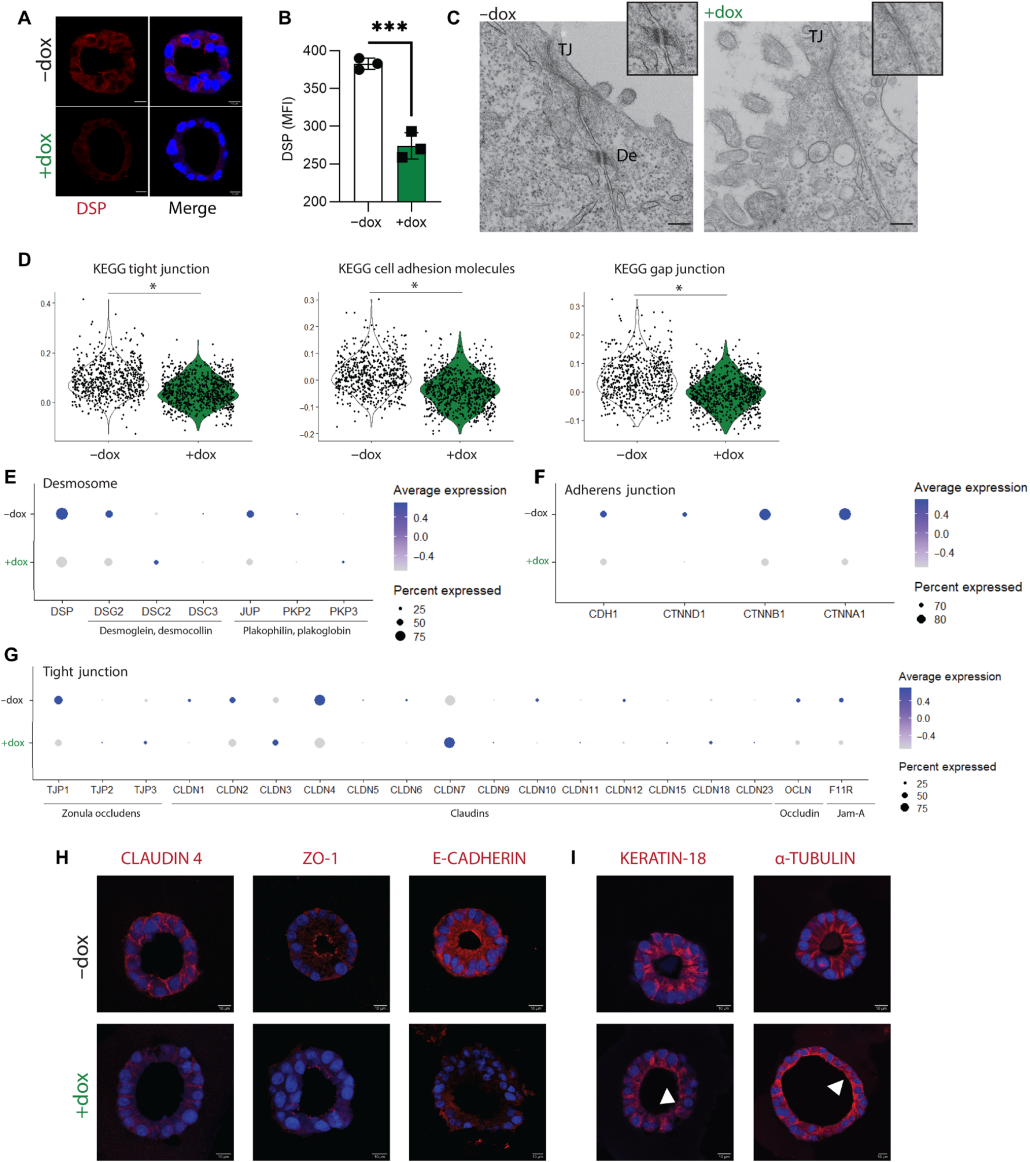
*DSP*-kd disrupts cell-cell junctions and cytoskeletal organization. CRISPRi-iAT2s were transduced with DSP gRNA and treated without (−dox) or with (+dox) doxycycline. (**A**) Immunofluorescence staining of desmoplakin (DSP). DSP, red; nuclei, blue; scale bar, 10 μm. (**B**) Flow cytometry analysis of DSP expression, represented by mean fluorescence intensity (MFI). *n* = 3 experimental replicates of independent wells of a differentiation; error bars represent SD. Statistical significance was determined by unpaired, two-tailed Student’s *t* test; ***P < 0.001. (**C**) Transmission electron microscopy images showing tight junctions (TJ) and desmosomes (De); scale bar, 200 nm. (**D**) Violin plots showing module scores for KEGG (Kyoto Encyclopedia of Genes and Genomes) tight junction, cell adhesion molecules, and gap junction. (**E**) Dot plots showing expression of genes associated with desmosome, (**F**) adherens junction, and (**G**) TJ. Genes that were not expressed by iAT2s were excluded from dot plots. (**H**) Immunofluorescence staining of claudin-4, ZO-1, and E-cadherin. (**I**) Immunofluorescence staining of keratin-18 and α-tubulin. Arrows indicate disorganized filaments or tubules. Protein of interest, red; nuclei, blue; scale bar, 10 μm.

DSP is found in desmosomes, cell junctions that provide structural integrity to tissues that experience mechanical stress, such as the skin, heart, and lung. DSP functions in the desmosome as a molecular link that tethers desmosomal cadherins to intermediate filaments (IFs) (*33*). Immunofluorescence and flow cytometry staining for DSP protein, encoded by *DSP*, demonstrated significantly reduced levels of DSP by CRISPRi-knockdown in iAT2s (Fig. 5, A and B). We used transmission electron microscopy (TEM) to visualize the ultrastructure of iAT2s; while tight junctions (TJs) were clearly visible, we found depletion of functional desmosomes in *DSP*-kd cells (Fig. 5C). Gene set enrichment analysis (GSEA) of differentially expressed genes revealed that pathways enriched in control (−dox) iAT2s included those involved in TJ, gap junction, and cell adhesion molecules (Fig. 5D and fig. S3, D and E). We next explored expression of key genes expressed in TJs, adherens junctions (AJs), or desmosomes. The expression of key desmosome genes (*DSP*, *DSG2*, *DSC3*, *JUP*, and *PKP2*) and AJ genes (*CDH1*, *CTNND1*, *CTNNB1*, and *CTNNA1*) was decreased in *DSP*-kd iAT2s (+dox) compared to control iAT2s (−dox) (Fig. 5, E and F). The expression of many TJ genes, including zona occludens (*TJP1*), claudins (*CLDN1* and *CLDN4*), occludin (*OCLN*), and JAM-A (*F11R*), was also down-regulated in the *DSP*-kd iAT2s (Fig. 5G). We next used immunofluorescence to investigate protein expression and confirmed that ZO-1 (encoded by *TJP1*), claudin-4, and E-cadherin (encoded by *CDH1*) expression was decreased in *DSP*-kd iAT2s compared to control (Fig. 5H). In addition to tethering IFs (*33*), DSP is also known to play a role in organizing microtubules in epidermal cells (*34*). Using immunofluorescence, we observed that the organization of both IFs (keratin-18) and microtubules (α-tubulin) was substantially perturbed in *DSP*-kd iAT2s (Fig. 5I), suggesting a pervasive disruption on all cellular junctions by *DSP*-kd in iAT2s. Together, these data suggest that DSP is required for the formation of desmosomes, TJs, AJs, and associated cytoskeletal organization in iAT2s.

### DSP expression regulates mitochondrial fatty acid oxidation

**Fig. 6.**
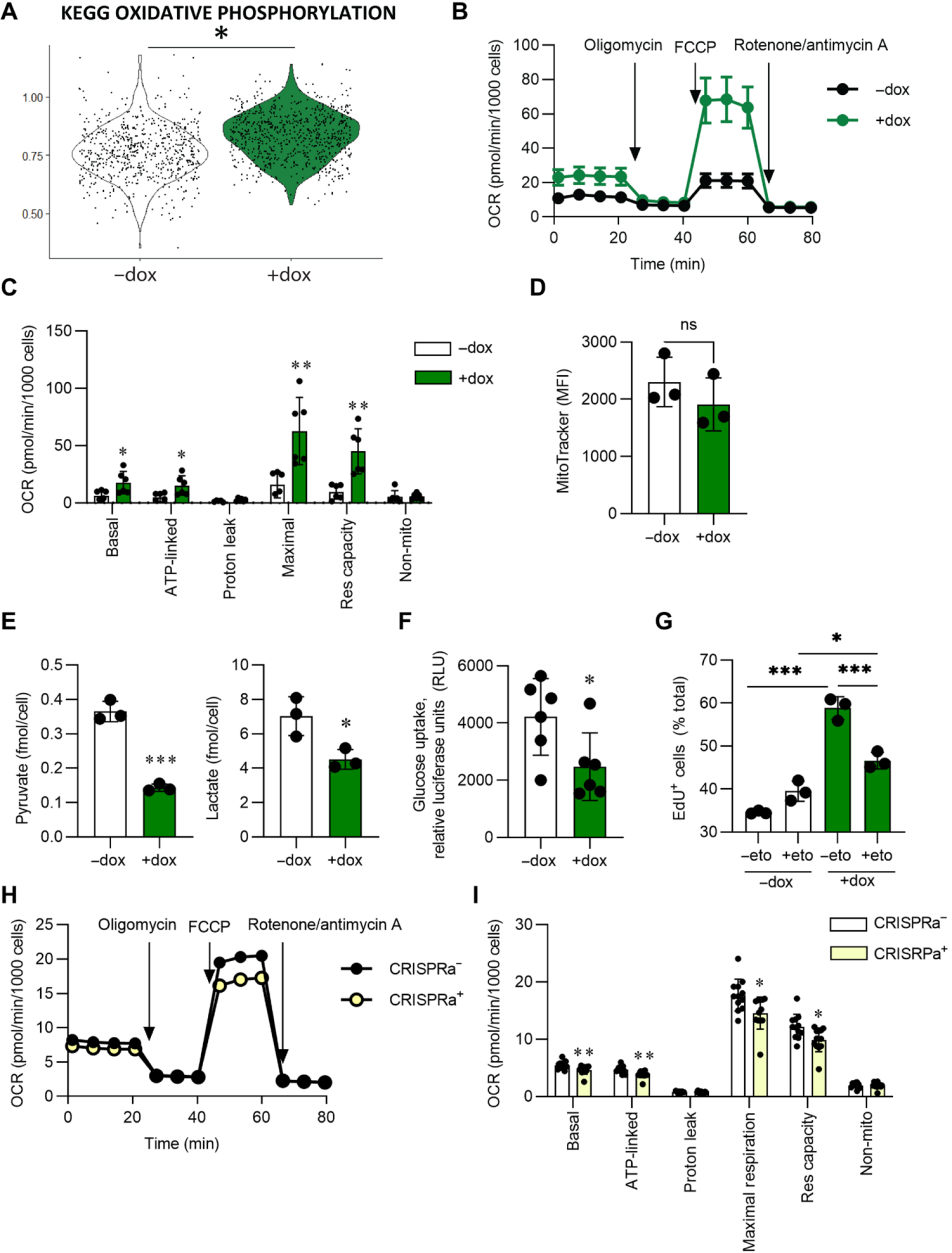
DSP expression influences mitochondrial respiration. CRISPRi-iAT2s were transduced with DSP gRNA and treated without (−dox) or with (+dox) doxycycline (**A** to **G**). (A) Module score for KEGG oxidative phosphorylation of scRNA-seq. (B and C) Basal oxygen consumption rate (OCR) was measured, followed by injection of oligomycin, FCCP, and rotenone + antimycin A, as indicated. Data were normalized by cell count after the assay was complete. *n* = 6 technical replicates of a differentiation. (D) MitoTracker staining was quantified by flow cytometry, represented by MFI; *n* = 3 experimental replicates of independent wells of a differentiation. (E) Pyruvate or lactate was measured in the supernatant and normalized by cell count; *n* = 3 experimental replicates of independent wells of a differentiation. (F) Glucose uptake was determined using 2-DG and measuring luciferase; *n* = 3 experimental replicates of independent wells of two differentiations. (G) Cells were treated with 10 μM etomoxir or vehicle for 21 days. One day before harvest, cells were incubated with EdU. EdU incorporation was measured by flow cytometry. *n* = 3 experimental replicates of independent wells of a differentiation. (**H** and **I**) iAT2s were transduced with DSP gRNA ± CRISPRa lentivirus. Basal OCR was measured, followed by injection of oligomycin, FCCP, and rotenone + antimycin A, as indicated. Data were normalized by cell count after the assay was complete. *n* = 10 technical replicates of a differentiation. All error bars represent SD. Statistical significance was determined using unpaired, two-tailed Student’s *t* test or a one-way ANOVA with a Tukey multiple comparison test; *P < 0.05, **P < 0.005, and ***P < 0.001.

We next identified pathways that were up-regulated by scRNA-seq following CRISPRi-knockdown of *DSP* in iAT2s. GSEA revealed that the most enriched pathways were related to metabolism, including oxidative phosphorylation, peroxisome, and fatty acid metabolism (Fig. 6A and fig. S4A). To evaluate mitochondrial function, we first measured the oxygen consumption rate (OCR) of iAT2s and found that *DSP*-kd iAT2s exhibited significantly increased basal, adenosine triphosphate (ATP)–linked, maximal, and spare respiratory capacity compared with control iAT2s (Fig. 6, B and C), consistent with our transcriptomic findings. We did not identify differences in total mitochondria by MitoTracker staining (Fig. 6D), suggesting that increased OCR was not the result of increases in mitochondrial quantity. Of note, extracellular acidification rate (ECAR) was not significantly altered between control or *DSP*-kd iAT2s, suggesting that glycolysis and/or pyruvate oxidation to CO_2_ were likely not affected by *DSP*-kd (fig. S4B). The OCR of iAT2s transduced with NT gRNA and treated with dox was unaffected (fig. S4, C and D). To understand the mechanism underlying increased respiration in *DSP*-kd iAT2s, we measured lactate levels and found decreased levels in *DSP*-kd cell supernatants, indicating that the cells were not favoring glycolysis to lactate (Fig. 6E). We additionally found that glucose uptake was decreased in *DSP*-kd iAT2s (Fig. 6F). The lack of an increase in ECAR, together with decreased glucose uptake and lower pyruvate and lactate, suggests that increased mitochondrial respiration was fueled by nutrients other than glucose. As fatty acid oxidation (FAO) can inhibit glucose uptake and oxidation (*35*), we hypothesized that *DSP*-kd iAT2s might preferentially use FAO to meet increased ATP demand induced by elevated increased proliferation. To investigate this possibility, we treated cells with the CPT-1 inhibitor etomoxir to block acyl–coenzyme A transport into the mitochondria and thereby inhibit FAO (*36*). As we had observed previously, *DSP*-kd iAT2s were again more proliferative compared to control iAT2s as measured by EdU incorporation; treatment with etomoxir, however, significantly attenuated the increase in cell proliferation induced by *DSP*-kd iAT2 (Fig. 6G). Last, we measured mitochondrial oxidative function in iAT2s overexpressing *DSP* using CRISPRa and found significantly decreased basal, ATP-linked, maximal, and spare respiratory capacity and a concomitant increase in pyruvate, lactate, and glucose uptake compared with control iAT2s (Fig. 6, H and I, and fig. S4, E to G). Together, these data suggest that *DSP* expression modulates mitochondrial function and FAO.

### DSP-kd modulates ERK-MAPK signaling to regulate proliferation in iAT2s

**Fig. 7.**
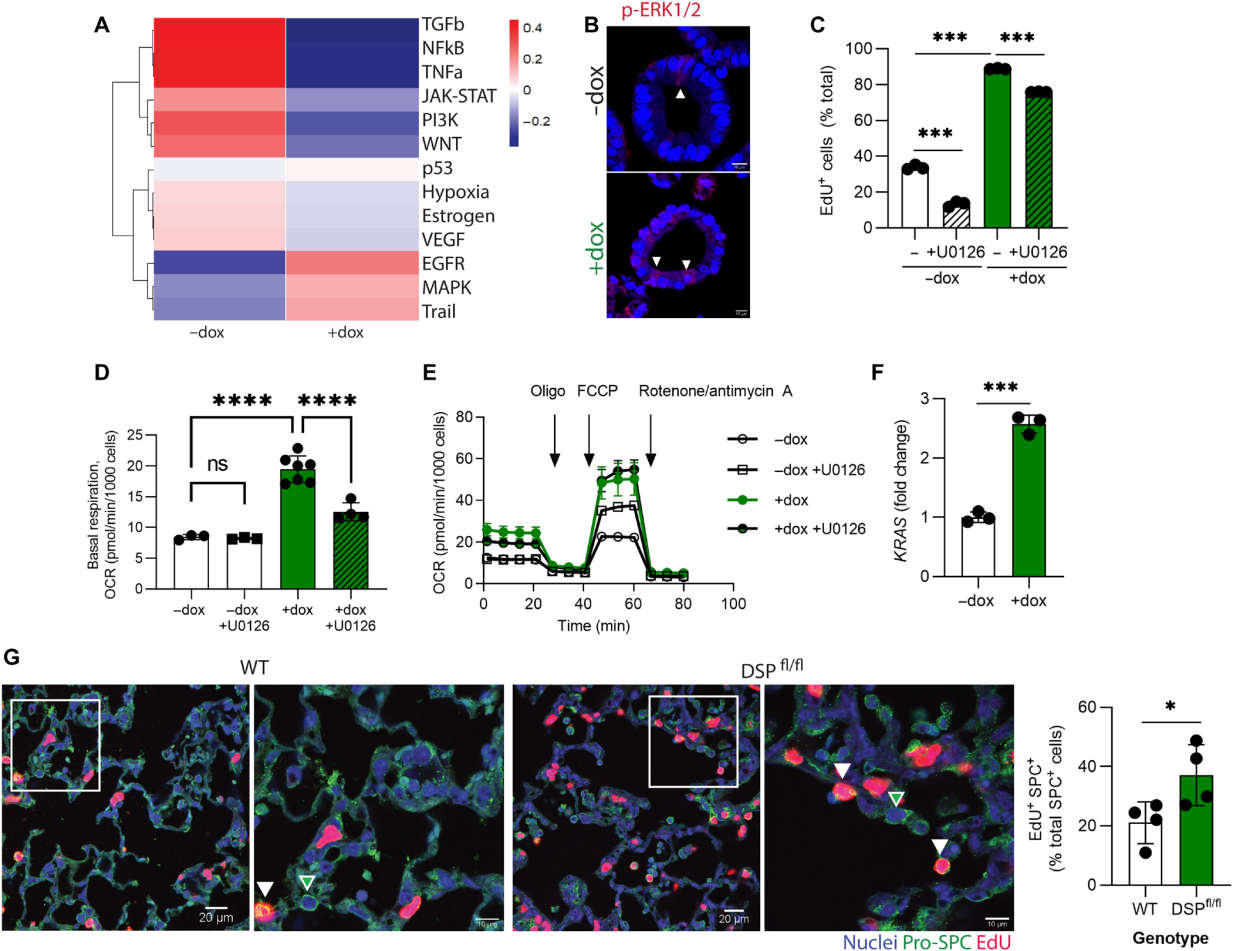
*DSP*-kd regulates proliferation through ERK-MAPK. CRISPRi-iAT2s were transduced with DSP gRNA and treated without (−dox) or with (+dox) doxycycline. (**A**) PROGENγ (Pathway RespOnsive GENes) of scRNA-seq. (**B**) Immunofluorescence of phosphorylated (p)-ERK1/2 (red); scale bar, 10 μm. (**C**) Cells were treated with U0126 for 3 days and pulsed with EdU for 24 hours, and EdU was measured by flow cytometry. *n* = 3 experimental replicates of independent wells of a differentiation. (**D** and **E**) Cells were treated with U0126 for 7 days, and then basal OCR was measured, followed by injection of oligomycin, FCCP, and rotenone + antimycin A, as indicated. Data were normalized by cell count after the assay was complete. (**F**) KRAS expression was measured by qRT-PCR; *n* = 3 experimental replicates of independent wells of a differentiation. (**G**) Nkx2.1-Cre mice were crossed with Dsp^fl/fl^ mice. Mice were inoculated with 200 PFU of influenza (IAV) and injected with EdU 1 day before sacrifice at 10 days after infection. Pro-SPC (green), EdU (red), and nuclei (blue) were stained for immunofluorescence. Green arrows denote EdU- Pro-SPC^+^ cells; white arrows denote EdU^+^ Pro-SPC^+^ cells. EdU^+^ SPC^+^ cells were quantified in a blinded manner and normalized to total SPC^+^ cells. *n* = 4 mice per group.

Given that decreased *DSP* expression is associated with heightened proliferation of iAT2s, we next sought to understand the molecular pathways underpinning this finding. To do so, we inferred pathway activity in the scRNA-seq dataset of control and *DSP*-kd iAT2s using PROGENy (Pathway RespOnsive GENes, an R package for inference of pathway activity from gene expression) (*37*, *38*). We found that Wnt, transforming growth factor–β (TGF-β), and tumor necrosis factor–α (TNF-α) pathways were down-regulated in *DSP*-kd iAT2s, while epidermal growth factor receptor (EGFR) and mitogen-activated protein kinase (MAPK) signaling were up-regulated (Fig. 7A). MAPK signaling plays an important role in regulating proliferation (*39*), and dysregulated MAPK has previously been identified in the context of DSP depletion in cardiac and skin cells (*40*, *41*). We found that expression of nuclear phosphorylated extracellular signal-regulated protein kinase (p-ERK1/2) was elevated in *DSP*-kd iAT2s compared to control iAT2s (Fig. 7B). To further investigate a role for MAPK signaling in the absence of *DSP* in iAT2s, we treated cells with inhibitors specific to each MAPK pathway [ERK, p38, and c-Jun N-terminal kinase (JNK)]. We found that inhibition of ERK signaling using the MAPK kinase 1/2 (MEK1/2) inhibitor U0126 significantly reduced proliferation of control iAT2s, consistent with a known role for EGFR/KRAS signaling in AT2 proliferation (Fig. 7C) (*42*, *43*). Moreover, ERK inhibition significantly dampened the elevated proliferation state of DSP-kd iAT2s (Fig. 7C). p38 inhibition (using SB205380) had no effect on iAT2 proliferation, while JNK inhibition (using SP600125) did decrease proliferation but likewise induced apoptosis, as measured by active caspase 3 staining (fig. S5, A and B). We next measured mitochondrial oxygen consumption to determine whether the reduction in iAT2 proliferation induced by inhibiting ERK signaling would prevent the increase in mitochondrial function. As expected, we observed significantly lower basal respiration in U0126-treated *DSP*-kd iAT2s but no change in maximal respiration, consistent with respiration changing mostly because of a change in ATP demand (Fig. 7, D and E). To understand why reduced *DSP* resulted in an increase in ERK signaling, we next measured expression of the upstream regulator *KRAS* and found it to be elevated in the DSP-kd iAT2s (Fig. 7F). Conversely, *DSP* overexpression decreased *KRAS* expression (fig. S5C). Collectively, these results suggest that the reduction of *DSP* modulates proliferation of iAT2s in part by inducing ERK signaling.

We next investigated whether DSP regulates AT2 proliferation in vivo*. Dsp*^fl/fl^ mice were crossed with *Nkx2.1*-Cre mice to generate adult mice lacking *Dsp* in Nkx2.1^+^ lung epithelial cells. AT2s are quiescent in the homeostatic lung, and we found no difference in proliferation of AT2s in uninjured *Dsp*^fl/fl^ mice. There were likewise no differences observed in alveolar size or in the number or distribution of lung epithelial cells (Nkx2-1^+^) or AT2s (pro-SPC^+^) in the absence of injury (fig. S5, D and E). We therefore inoculated mice with influenza A (IAV) to cause lung injury and prompt AT2 proliferation (*44*). Nine days after infection, we injected EdU to label dividing cells before harvesting lung tissue 24 hours later. While IAV infection induced epithelial proliferation in both wild-type (WT) and mutant mice, we found a significantly higher percentage of EdU labeling in AT2s from mice lacking Dsp in the epithelium (*Dsp*^fl/fl^) (Fig. 7G). Moreover, we identified fewer claudin-4^+^ AT2s in *Dsp*^fl/fl^ mice (fig. S5F), consistent with our findings in iAT2s.

### Loss of DSP mediates iAT2 migration, fibrosis, and cigarette smoke responses

**Fig. 8.**
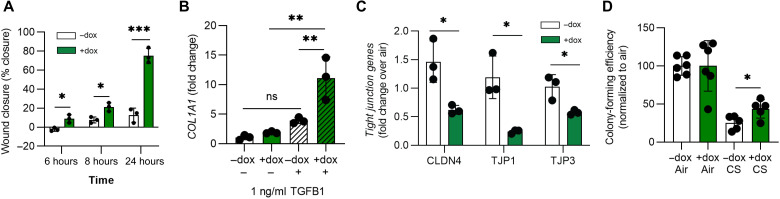
DSP expression alters the cellular response to injury. (**A**) Cells were plated in 2D and allowed to reach confluence before a scratch wound was made. Wound closure was calculated as a percentage of the initial wound over a 24-hour period. *n* = 3 experimental replicates of independent wells of a differentiation. (**B**) Cells were treated with TGF-β1 (1 ng/ml) for 48 hours before collecting RNA, and COL1A1 expression was measured by qRT-PCR. *n* = 3 experimental replicates of independent wells of a differentiation. (**C**) Cells were plated at ALI and then exposed to 5% cigarette smoke in a gas phase or humidified air. Cells were collected 2 hours after smoke exposure, and CLDN4, TJP1, and TJP3 were measured by qRT-PCR. *n* = 3 experimental replicates of independent ALIs of a differentiation. (**D**) Cells were exposed to 5% cigarette smoke in a gas phase or humidified air while plated at ALI and then replated in 3D Matrigel to reform alveolospheres over 2 weeks. Colony-forming efficiency was calculated relative to air-exposed cells. *n* = 3 experimental replicates of independent wells of two differentiations. All error bars represent SD. Statistical significance was determined using unpaired, two-tailed Student’s *t* test or a one-way ANOVA with a Tukey multiple comparison test; *P < 0.05, **P < 0.005, and ***P < 0.001.

Genetic variants associated with *DSP* have been implicated in both COPD and pulmonary fibrosis (*7*). To investigate how DSP might contribute to the pathogenesis of these diseases, we next evaluated the response of *DSP*-kd iAT2s to commonly applied stimuli with potential disease relevance. First, we performed scratch-wound assays (*45*) to assess the migratory capacity of iAT2s. We found that *DSP*-kd iAT2s more rapidly closed the wound compared to control iAT2s (Fig. 8A). Next, we explored how iAT2s would respond to a low dose of the profibrotic cytokine TGF-β1 that has been implicated in the pathogenesis of pulmonary fibrosis (*46*). Forty-eight hours after exposure, we found that *DSP*-kd iAT2s significantly up-regulated expression of extracellular matrix genes, *COL1A1*, *COL2A1*, and *FN1*, compared to control iAT2s (Fig. 8B and fig. S6A). Last, we exposed iAT2s to the inhaled stimulus common to COPD and pulmonary fibrosis, cigarette smoke. To do so, we plated iAT2s at air-liquid interface (ALI) to allow exposure to gas-phase cigarette smoke (5% smoke by volume) versus humidified room air. We have previously found that iAT2s cultured at ALI exit the cell cycle and form TJs (*26*), which are known to be disrupted in airway epithelium in response to smoke exposure (*47*). Two hours after cigarette smoke exposure, we collected cells for qRT-PCR or replated the cells in Matrigel. Compared to smoke-exposed control iAT2s, we found that smoke-exposed *DSP*-kd iAT2s had significantly reduced expression of TJ genes, such as *CLDN4*, *TJP1*, and *TJP3* (Fig. 8C). Next, we assessed colony-forming efficiency in iAT2s exposed to either smoke or air when replated in three-dimensional (3D) conditions. We found that *DSP*-kd iAT2s retained a greater capacity than control iAT2s to form spheres following smoke exposure and increased expression of the proliferation gene *MKI67* (Fig. 8D and fig. S6B). Last, we exposed iAT2s overexpressing *DSP* via CRISPRa to cigarette smoke and found that increased *DSP* expression was associated with increased expression of TJ genes and decreased *MKI67* expression following injury (fig. S6, C and D). Collectively, these data suggest that DSP constrains iAT2 migratory capacity and response to profibrotic stimuli and likewise modulates TJ formation and proliferation in the context of cigarette smoke–induced injury.

## DISCUSSION

In this study, we developed a CRISPRi iPSC platform to iteratively knock down genes of interest in differentiated lung epithelial cells and interrogate “gene × environment” interactions. We found that knockdown of multiple genes implicated in COPD by GWAS altered the AT2 phenotype as read out by differentiation, proliferation, and maturation of the AT2 program. On the basis of the results of this initial screen, we performed a detailed investigation of one gene, *DSP*, and found that in addition to regulating desmosome formation, *DSP* also influenced TJs, AJs, and cytoskeletal organization. We also found that DSP-kd led to increased mitochondrial activity to accommodate hyperproliferation of AT2s driven by ERK-MAPK.

Functional validation of GWAS-implicated genes requires both identification of the cell type affected in vivo and selection of a model system capable of faithfully recapitulating the biology of that cell type. GWAS variants fall in noncoding regions, making it likely that the regulation of gene expression is tissue and cell specific (*20*), further highlighting the importance of cell/model selection. Expression of genes implicated by GWAS is highly enriched in AT2s (*11*, *15*), a cell type that has been implicated in both the inception and the progression of COPD (*13*, *14*). The amenability of iPSCs to gene editing allowed us to generate a pure population of cells stably expressing an inducible CRISPRi construct. We elected to use CRISPRi rather than a CRISPR-knockout approach for our studies in part because GWAS variants typically modulate gene expression but rarely result in complete loss of gene function. We have likewise found that some genes implicated by GWAS are essential to differentiation of lung progenitors from human iPSCs, precluding the application of a knockout approach to an undifferentiated cell population for the purposes of interrogating gene function in their differentiated progeny.

Each of the genes we interrogated affected at least one iAT2 phenotype in our initial screen. *TGFB2* had no effect on differentiation or surfactant gene expression, but regulated iAT2 proliferation, consistent with the known role of TGFBs in inhibiting proliferation (*48*). Loss of *HHIP* or *SOX9* affected surfactant gene expression but no other measured phenotype. The expression pattern of *HHIP* differs among species: In mice, *Hhip* is expressed in myofibroblasts, whereas the human ortholog *HHIP* is expressed exclusively by AT2s in the adult lung (*17*), further highlighting the importance of studying these genes in relevant human cell types. *RBMS3* and *ADGRG6* did not affect iAT2 differentiation capacity but regulated expression of surfactants and iAT2 proliferation. The role that *RBMS3* or *ADGRG6* may play in AT2s has not been explored to date; these results suggest that further studies are warranted. Last, *DSP* and *FAM13A* both affected each iAT2 phenotype that we tested, though in some cases with opposing effects. For instance, *DSP*-kd increased iAT2 differentiation and proliferation, whereas *FAM13A*-kd decreased both. Of note, we have previously found that *FAM13A*-kd increased 16HBE cell proliferation (*49*), suggesting that the influence of FAM13A on proliferation is cell type dependent. Intriguingly, SNPs for *DSP* and *FAM13A* are associated with both COPD and pulmonary fibrosis but with opposite risk alleles (*7*), suggesting a potential mechanistic link between the two diseases and inviting further study to understand the contribution of these genes in particular.

DSP is an essential component of the desmosome. We found that functional desmosomes with anchored IFs failed to form in the context of *DSP*-kd, an observation previously made in skin (*50*) but not intestine (*51*). Desmosomal proteins are known to interact with TJs and AJs (*52*), and DSP itself directly interacts with many components of both junctional complexes (*53*). We found that in iAT2s, *DSP*-kd affected the expression of both TJ and AJ components, as well as organization of IFs and microtubules, significantly overlapping with previous observations in keratinocytes (*34*, *50*). DSP has been shown to suppress proliferation in immortalized cell lines (*54*, *55*), and we now report that the reduction of *DSP* alters proliferation of AT2s, both in vitro and in vivo. The heightened proliferation triggered by *DSP*-kd occurred in part through expression of *KRAS* and p-ERK1/2. DSP-kd in cardiomyocytes similarly leads to ERK1/2 activation via KRAS (*41*); however, the mechanisms through which DSP represses KRAS and subsequent ERK signaling remain unknown. In addition, our scRNA-seq data and subsequent analyses demonstrated an increase in mitochondrial respiration in DSP-kd iAT2s; follow-up experiments suggested that this increase was in part driven by increased proliferation triggered by reduction of *DSP*. Of note, similar to a subset of B cell lymphomas (*56*), AT2s use mitochondrial FAO to support proliferation, rather than glycolysis to lactate. The reason for this preference is unclear, but the advantage of fatty acid utilization is that it is the nutrient providing the most ATP per molecule of nutrient. Last, as variants in *DSP* are common to both COPD and pulmonary fibrosis (*7*), we applied injury models including cigarette smoke exposure, a risk factor for both diseases, and found that *DSP* in iAT2s mediates responses to wounding, profibrotic stimuli, and cigarette smoke injury. Together, our results suggest potential mechanisms through which DSP could contribute to the pathogenesis of both diseases. As increased *DSP* expression is associated with elevated risk for COPD (*57*), these could include decreases in AT2 proliferation and maturation following injury, leading to aberrant repair.

A limitation of our study is that our iAT2 cultures are composed of a single cell type when multiple epithelial, immune, and stromal cells interact in the lung alveolus and have been implicated in the pathogenesis of COPD. For this reason, we validated our findings in vivo using mouse genetic models, which similarly revealed that loss of lung epithelial Dsp results in augmented AT2 proliferation in the context of adult lung tissues in vivo. While the lack of mesenchymal or AT1 cells in our in vitro reductionist model system allowed us to isolate AT2-intrinsic responses, further studies in other model systems or advances in iPSC-directed differentiation protocols will be needed to identify potential effects of AT2 *DSP* expression on AT1s, their neighboring cells in the alveolus. We biased our gene selection approach to prioritize genes implicated by GWAS in COPD that are expressed in both primary AT2s and iAT2s and therefore excluded potentially disease-relevant genes that could be explored in other cell types, such as basal or secretory cells. Last, GWAS identify variants rather than genes or cell types and require use of complementary information, such as gene expression and overlap of open chromatin. In this case, strong evidence in the form of a lung-specific expression quantitative trait locus (eQTL) supports *DSP* as the causal gene implicated by GWAS at rs2076295 (*7*, *57*, *58*).

In conclusion, we applied CRISPRi in differentiated human iPSCs to interrogate the function of COPD GWAS genes in disease-relevant lung epithelial cells. We observed that multiple genes had an impact on at least one aspect of AT2 biology (differentiation, maintenance/maturation of AT2 transcriptional program, and proliferation). Using this system, we characterized the functional significance of DSP in AT2s and found that, in addition to contributing to formation of cell-cell junctions, DSP modulated proliferation and mitochondrial respiration through the ERK-MAPK pathway as well as the iAT2 response to injury. In future studies, through the application of alternate directed differentiation protocols, the iPSC-CRISPRi platform featured here could be applied to investigate the functional significance of GWAS genes in other cell types.

## MATERIALS AND METHODS

### Derivation and maintenance of iPSC lines

iPSCs were maintained in feeder-free conditions in mTeSR1 medium (STEMCELL Technologies, 05850) on hESC-Qualified Matrix (Corning, 8774552), using Gentle Cell reagent for passaging (STEMCELL Technologies, 07174). For targeting, iPSCs were replated on mitotic inactivated mouse embryonic fibroblast (MEF) feeder cells in human iPS medium [20% knockout serum (Life Technologies), GlutaMAX (Life Technologies), nonessential amino acids (Life Technologies), fibroblast growth factor 2 (FGF2) (10 ng/ml; R&D Systems), 100 μM β-mercaptoethanol (Life Technologies), and Primocin (InvivoGen) in Dulbecco’s modified Eagle’s medium/D12 (Life Technologies)]. iPSCs on feeders were passaged with collagenase type 4 (1 mg/ml; Gibco). All iPSCs displayed a normal karyotype when analyzed by G-banding (Cell Line Genetics). Additional details pertaining to iPSC derivation, characterization, and culture are available for free download at https://crem.bu.edu/cores-protocols/. iPSCs used here are available upon request from the CReM iPSC repository at https://stemcellbank.bu.edu.

### Targeting CRISPRi construct to AAVS1 locus in iPSCs

iPSC lines targeted with CRISPRi constructs were BU3 NGST and RUES2 ST (*22*, *27*). pAAVS1-NDi-CRISPRi (Gen2) was a gift from B. Conklin (Addgene plasmid no. 73498; http://n2t.net/addgene:73498; RRID:Addgene_73498) (*24*), and AAVS1 targeting constructs (AAVS1-TALEN-L and AAVS1-TALEN-R) were a gift from D. Huangfu (Addgene plasmid no. 59025; http://n2t.net/addgene:59025; RRID:Addgene_59025 and Addgene plasmid no. 59026; http://n2t.net/addgene:59026; RRID:Addgene_59026) (*59*). iPSCs were preincubated with Y-27632 dihydrochloride (Tocris) for 3 hours and then dissociated to single-cell suspension with Gentle Cell reagent. Cells were nucleofected (Lonza Amaxa, program CB150) with 3 μg of pAAVS1-NDi-CRISPRi (Gen2), 1 μg of AAVS1-TALEN-L, and 1 μg of AAVS1-TALEN-R in P3 solution (Lonza, V4XP-3024) and then replated on drug-resistant (DR4) MEFs. Integration of the CRISPRi construct (carrying a neomycin-resistant cassette) was selected starting 2 days later with geneticin (G418, InvivoGen). Selected colonies were manually picked 10 to 15 days later and screened further for integration while simultaneously expanding in larger culture format.

### PCR for AAVS1 integration

Genomic DNA (gDNA) was extracted by adding 20 μl of proteinase K buffer (1× PCR buffer + 100 μg/ml proteinase K) to half of a picked colony. PCR was performed using AccuPrime Pfx DNA Polymerase (Invitrogen). PCR products were analyzed by gel electrophoresis and Sanger sequencing (Genewiz). PCR primers were as follows: allele screen, TCTGGCTCCATCGTAAGCAAACCT (forward) and CCCCTATGTCCACTTCAGGA (reverse); integration screen, CGGGTCACCTCTCACTCCTTTCATTT (forward) and GGCCTTCCATCTGTTGCTGC (reverse).

### Southern blot

The probe was generated and labeled with [α-^32^P]dCTP (deoxycytidine triphosphate) immediately before use and purified by a G50 column (GE Healthcare). gDNA was extracted using the Qiagen gDNA Extraction Kit; 10 μg of gDNA was then digested with 20 U of SphI (NEB). Digested gDNA was run on a 0.8% agarose gel overnight and then transferred to a Hybond-N membrane overnight (GE Healthcare). The membrane was prehybridized in Rapid-Hyb buffer (GE Healthcare) and then hybridized with the radiolabeled probe at 65°C for 4 hours. Following washes with SSC buffer, the membrane was exposed to film overnight at −80°C. WT (no integration) had a 6500–base pair (bp) band compared with CRISPRi integrated lines, which had a 3500-bp band. Primers to generate the probe were AGGTTCCGTCTTCCTCCACT (forward) and GTCCAGGCAAAGAAAGCAAG (reverse).

### gRNA design and synthesis

gRNAs were designed using the online gRNA design tool crispor.org to target the TSS of genes of interest (−50 to +200 bp) (*24*, *60*). To accomplish sustained knockdown, gRNAs were cloned into a lentiviral vector. pLV GG hUbC-dsRED, ph7SK-gRNA, phU6-gRNA, pmU6-gRNA, and phH1-gRNA were a gift from C. Gersbach (Addgene plasmid no. 84034; http://n2t.net/addgene:84034; RRID:Addgene_84034, Addgene plasmid no. 53189; http://n2t.net/addgene:53189; RRID:Addgene_53189, Addgene plasmid no. 53188; http://n2t.net/addgene:53188; RRID:Addgene_53188, Addgene plasmid no. 53187; http://n2t.net/addgene:53187; RRID:Addgene_53187, Addgene plasmid no. 53186; http://n2t.net/addgene:53186; RRID:Addgene_53186). We modified the pLV GG hUbC-dsRED plasmid to express the EBFP2 reporter (pLV GG hUbC-EBFP2). gRNAs were cloned to be expressed from individual RNA polymerase III promoters and then assembled to the lentiviral vector by Golden Gate cloning. The sequences for all gRNA are included in Table 1 and all associated lentiviral backbone plasmids created for gRNA delivery are deposited at Addgene.

**Table 1.** Guide RNA (gRNA) sequences used in this study.

**Gene target**	**Protospacer sequence**
NT	GTATTACTGATATTGGTGGG
SFTPC g1	GATGGATGTGGGCAGCAAAG
SFTPC g2	ACCTGCAGCAAGATGGATGT
SFTPC g3	ACGCAACCACACTCACCGGC
FAM13A g1	AGCTGCTCTTCGCTTACCTT
FAM13A g2	CTAGCCATCTGTGTAAGTAT
FAM13A g3	GTCGTTTTAATATTTTCGTC
DSP g1	CGGGTGTCACCGACGCGCTC
DSP g2	CAGAGGAGCTGCGTCGGAGC
DSP g3	ACCCTGGGAAGAAACCGGCC
DSP RNP gRNA	AGGATGTACTATTCTCGGCG
MFAP2 g1	ACTTACCAGGCAGGAATAGC
MFAP2 g2	GGGGGTAGCTCCTCTTATGT
MFAP2 g3	CTGAGGAGTAGGGTTAGTAG
MFAP2 g4	TTGTCACGACTTATGACCAG
RBMS3 g1	CCCCGAACACCATGTCACTC
RBMS3 g2	AGCAGCAACTAAGCTGTACA
RBMS3 g3	GAAGCTGATCTGCAAGGATT
RBMS3 g4	TTACGAACGCTGGCAATTGA
SOX4 g1	AAGAGGCCTGTTTCGCTGTC
SOX4 g2	GTTTCGCTGTCGGGTCTCTA
SOX4 g3	ACTCCTTAGTGCCGATTCCG
SOX4 g4	GAGCTACCGAGAGCGCTCGT
SOX9 g1	ATTCGCCTCCCCCCACTTGG
SOX9 g2	TAAGTGCTCGCCGCGGTAGC
SOX9 g3	GGAGCCGCTTGCTCCGCATC
SOX9 g4	CTCGAGTCCCCGAGCCGCCG
TGFB2 g1	CCCCGGCAAGATCGTGATGT
TGFB2 g2	AGCAGAAGGTTCGCTCCGAG
TGFB2 g3	AATATTAGCCTGACGGTCTA
TGFB2 g4	CTAAGCGAGCAATTCCACGT
HHIP g1	CTTCTTTTTAACTAGCGCGC
HHIP g2	GCGAGAAGCGGTGACGTCAA
HHIP g3	TGTTCACTTGTCCGTGTAAC
HHIP g4	AGGAACAGAAACGGCGACGG
ADGRG6 g1	CAGGGTTCACCTTGCGCCGT
ADGRG6 g2	ACCCTCCTTTACGCCGTTTC
ADGRG6 g3	GAGGATGATCTTGCGGCCAA
ADGRG6 g4	TACTGCGCCGAGGTCCCCTT

### Delivery of gRNA

For lentiviral delivery of gRNA to lung progenitors, lentivirus was added at a multiplicity of infection (MOI) of 10 with polybrene at day 12 (D12) of the differentiation. On D15 of the differentiation, cells were sorted for green fluorescent protein–positive (GFP^+^) (NKX2-1) and BFP^+^ (gRNA). For transduction of iAT2s, cells were dissociated to single-cell suspension and then resuspended with lentivirus (MOI 20 + polybrene + Y) and incubated in suspension for 4 hours before replating in 3D Matrigel. Transduced iAT2s were sorted for BFP 7 days after transduction.

### Lung-directed differentiation protocol and maintenance of iAT2s

Human PSC lines, including BU3 NGST CRISPRi, BU3 NGST, and RUES2 ST CRISPRi, underwent directed differentiation to generate iAT2s, as we have previously published (*22*). In brief, PSCs maintained in mTeSR medium were differentiated to definitive endoderm using the STEMdiff Definitive Endoderm Kit (STEMCELL Technologies, 05110), and definitive endoderm induction was confirmed by CXCR4^+^ cKit^+^, scored by flow cytometry. At the definitive endoderm stage, cells were dissociated with Gentle Cell Dissociation Reagent and replated on plates precoated with growth factor–reduced Matrigel (Corning). Cells were cultured for 72 hours in anteriorization medium (“DS/SB”) consisting of complete serum-free differentiation medium (cSFDM) base medium, as described (*22*) with 2 mM dorsomorphin (“DS”; Stemgent) and 10 mM SB431542 (“SB”; Tocris), with the first 24 hours also supplemented with 10 mM Y-27632 (Tocris). The cells were then cultured for an additional 9 to 10 days in lung progenitor induction medium (“CBRa”), consisting of cSFDM supplemented with 3 mM CHIR99021 (“C”; Tocris), recombinant human BMP4 (10 ng/ml; “B”; R&D Systems), and 100 nM retinoic acid (“Ra”; Sigma-Aldrich). On days 14 and 15 of differentiation, cells were sorted to isolate NKX2-1^+^ lung progenitors on a high-speed cell sorter (MoFlo Astrios). Once sorted, NKX2-1^+^ lung progenitors were resuspended in growth factor–reduced Matrigel (Corning, CB-40230) droplets and covered with distal differentiation medium (“CK + DCI”), which contains a base of cSFDM with 3 mM CHIR99021 (C), rhKGF (10 ng/m; “K”; R&D Systems), 50 nM dexamethasone (“D”; Sigma-Aldrich), 0.1 mM 8-bromoadenosine 3′,5′ cyclic monophosphate sodium salt (Sigma-Aldrich), and 0.1 mM 3-isobutyl-1-methylxanthine (IBMX; Sigma-Aldrich) (“CI”). To maintain pure cultures, cells were sorted when necessary to further enrich for NKX2-1^+^ cells expressing the SFTPC-tdTomato reporter. iAT2s were maintained in culture by refeeding every other day with CK + DCI medium and were serially passaged every 2 weeks at a density of 400 cells/μl, according to our previously published protocol (*22*, *61*, *62*). To knock down genes of interest in CRISPRi-targeted PSC lines, cells were maintained in either CBRa or CK + DCI medium containing 2 μM dox (Sigma-Aldrich, D9891). For specific experiments, iAT2 alveolospheres were treated with recombinant human TGF-β1 (1 ng/ml; R&D Systems, 7754-BH-005), (+)-etomoxir sodium salt hydrate (10 μM; Sigma-Aldrich, E1905-5MG), U0126 (10 μM; Tocris, 1144), SB203580 (20 μM; Calbiochem, 559389-1MG), or SP600125 (10 μM; Cell Signaling Technology, 8177), as indicated in the text.

### Reverse transcription quantitative PCR

Cells were collected and stored in Qiazol (Qiagen, 79306) before RNA extraction using the RNeasy Plus Mini Kit as per the manufacturer’s protocol (Qiagen, 74104). MultiScribe Reverse Transcriptase (Applied Biosystems, 4374967) was used to generate complementary DNA (cDNA). RT-qPCR was run for 40 cycles using predesigned TaqMan probes (Applied Biosystems, 4367846) or using custom-designed primers with SYBR Select Master Mix (Thermo Fisher Scientific, 4472908). The average Ct value for technical triplicates was calculated and normalized to 18*S* control. Fold change was determined over control cells using 2^ΔΔCt^. Biological replicates, as indicated for each figure legend, were run for statistical analyses. TaqMan (Thermo Fisher Scientific) probes used were as follows: *SFTPC (*Hs00161628_m1), *SFTPA1* (Hs00831305_s1), *SFTPA2* (Hs04195463_g1), *SLPI* (Hs00268204_m1), *NKX2-1* (Hs00968940_m1), *DSP* (Hs00950591_m1), *FAM13A* (Hs01596554_m1), *TGFB2* (Hs00234244_m1), *RBMS3* (Hs01104892_m1), *MFAP2* (Hs01027737_m1), *SOX4* (Hs04987498_s1), *SOX9* (Hs00165814_m1), *ADGRG6* (Hs01089210_m1), *HHIP* (Hs01011009_m1), *MKI67* (Hs04260396_g1), *CDH1* (Hs01023895_m1), *CLDN4* (Hs00533616_s1), *CLDN1* (Hs00221623_m1), *TJP1* (Hs01551876_m1), *TJP3* (Hs00274276_m1), *KRAS* (Hs00364284_g1), *COL1A1* (Hs00164004_m1), *COL2A1* (Hs00264051_m1), and *FN1* (Hs01549976_m1). Sybr primer sequences used were as follows: qPCR CAS9, CCGAAGAGGTCGTGAAGAAG (forward) and GCCTTATCCAGTTCGCTCAG (reverse); qPCR BACTIN, TTTTTGGCTTGACTCAGGATTT (forward) and GCAAGGGACTTCCTCTAACAAC (reverse).

### Flow cytometry and FACS

Single-cell suspensions were generated using 0.05% trypsin. Endoderm cells were stained for CXCR4 (MHCXCR404, Life Technologies) and c-Kit (CD11705, Life Technologies). To sort NKX2-1^+^ lung progenitors, cells containing an NKX2-1–GFP reporter were resuspended in sort buffer [Hanks’ balanced salt solution (Thermo Fisher Scientific), 2% fetal bovine serum, 10 μM Y-27632, and 10 μM calcein blue AM (Life Technologies)]. Cells without the NKX2-1–GFP reporter were stained with CD47-PerCPCy5.5 and CD26-PE (phycoerythrin) (mouse monoclonal, nos. 323110 and 323110, BioLegend) for 30 min on ice, washed with phosphate-buffered saline (PBS), and then resuspended in sort buffer. Further sorts were required to enrich for SFTPC^+^ iAT2s by sorting for SFTPC-tdTomato, or for lentivirus-gRNA by sorting for EBFP2, using Zombie NIR Fixable Viability dye (BioLegend, 423106) for live-dead discrimination. All cell sorts were performed on a high-speed cell sorter (MoFlo Astrios EQ, Beckman Coulter) at the Boston University Flow Cytometry Core Facility (FCCF).

For flow cytometry, cells were fixed with 4% paraformaldehyde (PFA) for 20 min at 37°C and then permeabilized with saponin buffer. Cells were stained for intracellular expression of DSP (Abcam, ab109445) or 200 nM MitoTracker Deep Red FM (Invitrogen, M22426). To determine EdU incorporation, cells were pulsed with 10 μM EdU for 24 hours (Invitrogen, C10424). Cells were then fixed and permeabilized with Click-iT fixative and permeabilization buffer and then stained for EdU using a Click-iT Plus EdU AF647 flow cytometry kit (Invitrogen), as per the manufacturer’s instructions.

Gating was based on isotype-stained controls (for antibody stains) or unstained controls (for EdU). Flow cytometry staining was quantified using Stratedigm S1000EXI and analyzed with FlowJo v10.6.2 (FlowJo, Tree Star Inc.). Fluorescence-activated cell sorting (FACS) plots shown represent single cells based on forward-scatter/side-scatter gating.

### Immunohistochemistry

iAT2 alveolospheres were fixed in 4% PFA for 20 min at 37°C and then immediately stained or dehydrated and embedded in paraffin blocks. Mouse lungs were fixed in 4% PFA overnight and then embedded in paraffin blocks. Before staining, paraffin-embedded sections were dewaxed and antigen retrieval was performed with heated citrate buffer. Samples were permeabilized with 0.3% Triton and blocked with 4% normal donkey serum (NDS). Primary antibodies were diluted in 4% NDS and incubated overnight at 4°C. Primary antibodies used were as follows: DSP (Abcam, ab109445), ZO-1 (Invitrogen, 61-7300), claudin-4 (Invitrogen, 32-9400), E-cadherin (Santa Cruz Biotechnology, sc-59778), keratin-18 (Invitrogen, MA5-12104), α-tubulin (Invitrogen, A11126), cleaved caspase-3 (Sigma-Aldrich, C8487), Pro-SFTPC (Seven Hills Bioreagents, WRAB-9337), NKX2-1 (Abcam, 76013), podoplanin (Invitrogen, 14-5381-82), and p44/42 MAPK (ERK1/2) (137F5) (Cell Signaling Technology, 4695). Sections were washed with 0.05% Tween and then incubated with fluorescent-conjugated secondary antibodies for 1 hour at room temperature. EdU in mouse sections was detected using the Click-iT EdU Alexa Fluor 647 Imaging Kit (Thermo Fisher Scientific, C10340). Samples were counterstained with Hoechst 33342. Cells were imaged with a Leica SP5 confocal microscope, and images were processed in ImageJ and Fiji.

### Western blot

Protein concentration was determined by bicinchoninic acid assay (Pierce) and then run on 4 to 12% bis-tris gels (Life Technologies). Protein was transferred to a polyvinylidene difluoride membrane using the iBlot system (Thermo Fisher Scientific). Membranes were blocked in 5% milk in tris-buffered saline and Tween 20 (TBS-T) and then probed with primary antibody [Cas9 antibody (7A9-3A3), Santa Cruz Biotechnology, sc-517386, or glyceraldehyde-3-phosphate dehydrogenase (GAPDH) antibody, Millipore, MAB374] overnight at 4°C. Following washing, membranes were incubated with horseradish peroxidase–conjugated secondary antibodies for 1 hour at room temperature, and then chemiluminescence was developed with SuperSignal West Dura Extended Duration Substrate (Thermo Fisher Scientific).

### Transmission electron microscopy

iAT2 alveolospheres were fixed for 3 hours at room temperature in 2% glutaraldehyde + 1% PFA in Na cacodylate buffer (pH 7.4). After washing, alveolospheres were postfixed overnight in 1.5% osmium tetroxide (Polysciences, catalog no. 0223D-10) at 4°C and block-stained in 1.5% uranyl acetate for 1 hour (Electron Microscopy Sciences, catalog no. 22400). After dehydration through graded acetones, tissue was infiltrated and embedded in Embed 812 Resin (Electron Microscopy Sciences, catalog no. 14120). Embedded samples were thin sectioned (70 nm), and grids were stained in 4% aqueous uranyl acetate for 10 min at room temperature followed by lead citrate for 10 min at room temperature. Electron microscopy was performed on a JEOL JEM-1400Flash TEM operated at 120 kV, and images were recorded on an AMT NanoSprint-43M-B Mid-Mount CMOS camera with a large 7915 × 5436 pixels format (captured images were 0.000386 μm per pixel).

### Single-cell RNA-seq

iAT2s (D184, 7 days after passage) were dissociated to single cells with 0.05% trypsin, and cells were incubated with Fc block (BioLegend, no. 422301) and stained with hashing antibodies (BioLegend, nos. 394631 and 394633). Live cells were sorted using Zombie NIR (BioLegend, no. 423106) on a MoFlo Astrios EQ (Beckman Coulter). Hashed cells were pooled 1:1 before capture. Live cells were captured, and libraries were prepared as per the 10x Genomics scRNA-Seq 3′v3.1 and hashtag oligonucleotide (HTO) protocols. Libraries were quantified by a Kapp kit, and GEX and HTO libraries were sequenced using an Illumina NextSeq 2000 instrument, pooled 50:1. The sequencing generated reads with 94% ≥ Q30. We used the Cellranger 3.0.2 pipeline to generate the fastq files and the count matrices (combining gene expression and antibody capture libraries for each sample). We then used Seurat (v 4.0.1) to further process and analyze the data. The HTODemux function was used to demultiplex the samples based on the hashing antibody capture assay. Each cell was classified as positive or negative for each HTO, and cells that were positive for more than one HTO were annotated as doublets. Cells with more than 15% of Unique Molecular Identifiers mapping to mitochondrial genes were filtered out, as well as cells with fewer than 800 genes detected. Potential doublets were identified and excluded (based on abnormally high number of genes detected in proportion to cell density, as per 10x Genomics recommendations). Data were normalized using the regularized negative binomial regression method (SCTransform function), and cell degradation (mitochondrial percentage) was regressed out. We then performed principal components analysis (PCA) on the sparse expression matrix and UMAP on the top 20 principal components. Clustering was computed with the Louvain algorithm at different resolutions, ranging from 1.5 to 0.05. Cell cycle stage and the enrichment in other molecular signatures were calculated using the scoring method from (*63*), as implemented in Seurat. Differential gene expression was determined by a log fold change of 0.25 with a Wilcoxon rank sum test. GSEA was performed using hypeR. scRNA-seq data are deposited at Gene Expression Omnibus (GEO): GSE189068. We also used Seurat to further analyze published datasets (GEO: GSE135893 and GSE168191) (*30*, *31*).

### Measurements of metabolic activity

OCR was measured using the Extracellular Flux Analyzer (Seahorse Bioscience). iAT2s were dissociated to single cell and plated in CKDCI+Y (+/−dox) into XFe96 Seahorse plate (coated with 2D Matrigel) at a density of 50,000 cells per well. OCR was measured the next day using Agilent Seahorse XF Base Medium (Agilent Technologies) supplemented with 25 mM glucose and 10 mM pyruvate. Oligomycin (2 μM;, Agilent), carbonyl cyanide-4-(trifluoromethoxy) phenylhydrazone (FCCP; 1.5 μM; Agilent), and antimycin A plus rotenone (both 1 μM; Agilent) were added over the course of the assay. Upon completion of the assay, the plate was fixed with 4% PFA, cells were stained with Hoechst, and each well was imaged. Cell counts were determined using ImageJ (analyze particles function), and OCR and ECAR were normalized by cell count.

Extracellular release of pyruvate and lactate in cell supernatants was measured as per the manufacturer’s instructions (MAK071 and MAK064, Sigma-Aldrich). Absolute pyruvate and lactate was normalized to total cell numbers per well.

Glucose uptake was measured using Glucose Uptake-Glo Assay (Promega, J1341). A total of 10,000 cells (in triplicate) per condition were resuspended in PBS and transferred in suspension to a 96-well plate. 2-Deoxyglucose (2-DG) was added, and luciferase was measured as per the manufacturer’s protocol (Promega).

### Scratch-wound assay

To assess cellular migration capacity, we performed a scratch-wound assay (*45*). In brief, 300,000 iAT2s were plated in CKDCI+Y on 2D Matrigel–coated 48-well plates and allowed to form a confluent monolayer overnight. A straight scratch was made through the center of the monolayer. Cells were imaged immediately (time, 0 hours) and then at 6, 8, and 24 hours. Cell migration was calculated as a percentage of scratch closure.

### Cigarette smoke exposure

To model the cellular response to cigarette smoke injury, iAT2s were plated at ALI, as previously described (*26*, *64*). University of Kentucky 3R4F reference cigarettes were preconditioned in a temperature- and humidity-controlled chamber for 48 hours in accordance with ISO 3402, as described (*26*). Cells were exposed to gas-phase combustible cigarette smoke using a Vitrocell VC1 in vitro smoke exposure system (Vitrocell Systems) following the ISO 3308 protocol. In brief, every 60 s, 35-ml puffs were drawn for 2 s, with a total of 32 puffs per exposure. Cigarette smoke was diluted with humidified (90%) room air to expose cultures to 5% (v/v) smoke. To study colony-forming efficiency after injury, we dissociated air or smoke-exposed ALIs with Accutase (Millipore-Sigma, A6964) and replated single cells in 3D Matrigel (400 cells/μl). Colony formation was monitored over 14 days and then imaged and quantified.

### CRISPR activation

For overexpression experiments, iAT2s were transduced with lentiviral-gRNA constructs (the same gRNA sequences used for CRISPRi) and sorted (based on BFP^+^) to purify gRNA-expressing cells. Cells were then transduced with lenti dCAS-VP64_Blast [a gift from F. Zhang (Addgene viral preparation no. 61425-LVC; http://n2t.net/addgene:61425; RRID:Addgene_61425)].

### CRISPR RNP

gRNAs were assembled by annealing CRISPR RNA (crRNA) (targeted to exon 1 of *DSP)* with trans-activating CRISPR RNA (tracrRNA) (IDT) in equimolar ratio at 95°C for 5 min, as per the manufacturer’s instructions. Ribonucleoprotein (RNP) complex was then assembled by mixing gRNA (120 pmol) with HiFi Cas9 Nuclease V3 (104 pmol) (IDT) and incubated at room temperature for 20 min. iAT2s were dissociated to single cells and then nucleofected with RNP complex + Cas9 electroporation enhancer (0.2 μM) in P3 solution (Lonza) using the EA104 program. Cells were then immediately replated in Matrigel. Note that this protocol results in mosaic-edited clones.

### Mouse experiments

*Nkx2-1*-Cre mice (C57BL6/J) were crossed with *Dsp^fl/fl^* mice (C57BL6/J). Mice were housed in Brigham and Women’s Hospital animal facility with a 12-hour light/dark cycle. This study was performed in accordance with the recommendations in the *Guide for the Care and Use of Laboratory Animals* of the National Institutes of Health. Tamoxifen-induced deletion of Dsp was initiated at 8 weeks of age after completion of alveolarization and lung maturation. At approximately 10 weeks old, Nkx-Cre^+/−^/Dsp^fl/fl^ or Nkx-Cre^+/−^/Dsp^WT^ mice were intranasally inoculated with 200 plaque-forming units (PFUs) of PR8 IAV. Mice were injected at 9 days after infection with EdU (10 mg/kg; Thermo Fisher Scientific) and then sacrificed at 10 days after infection. The lungs were harvested, perfused with PBS, and fixed in 4% PFA before embedding in paraffin. Note that Nkx2.1 is most highly expressed in AT2s, basal cells, and club cells (*65*).

### Statistics

Unpaired two-tailed Student’s *t* tests were used to compare two groups, one-way analysis of variance (ANOVA) with a Tukey multiple comparisons test was used to compare three or more groups, and details are provided in each figure legend. A *P* value of <0.05 was determined to be statistically significant, and *P* value annotations on graphs were annotated as follows: *P < 0.05, **P < 0.01, ***P < 0.001, ****P < 0.0001. Data are represented as means, with error bars representing SD.

### Study approval

All experiments involving the differentiation of human iPSC lines were performed with the approval of the Institutional Review Board of Boston University (protocol H33122). Mouse experiments were performed with the approval of the Institutional Animal Care and Use Committee (IACUC) at the Mass General Brigham Hospital.

## Notes

View/request a protocol for this paper from *Bio-protocol*.
