# Supplementary material for: CRISPR interference interrogation of COPD GWAS genes reveals the functional significance of desmoplakin in iPSC-derived alveolar epithelial cells
Source: Sci Adv. 2022 Jul 13;8(28):eabo6566. doi: 10.1126/sciadv.abo6566 (PMC9278866; doi:10.1126/sciadv.abo6566)
Supplement: Supplementary file 1 — Figs. S1 to S6 Table S1 [file sciadv.abo6566_sm.pdf]

Science Advances
AAAS

Supplementary Materials for

CRISPR interference interrogation of COPD GWAS genes reveals the functional significance of desmoplakin in iPSC-derived alveolar epithelial cells

Rhiannon B. Werder *et al.*

Corresponding author: Andrew A. Wilson, awilson@bu.edu

Sci. Adv. **8**, eabo6566 (2022)
DOI: 10.1126/sciadv.abo6566

This PDF file includes:

Figs. S1 to S6
Table S1

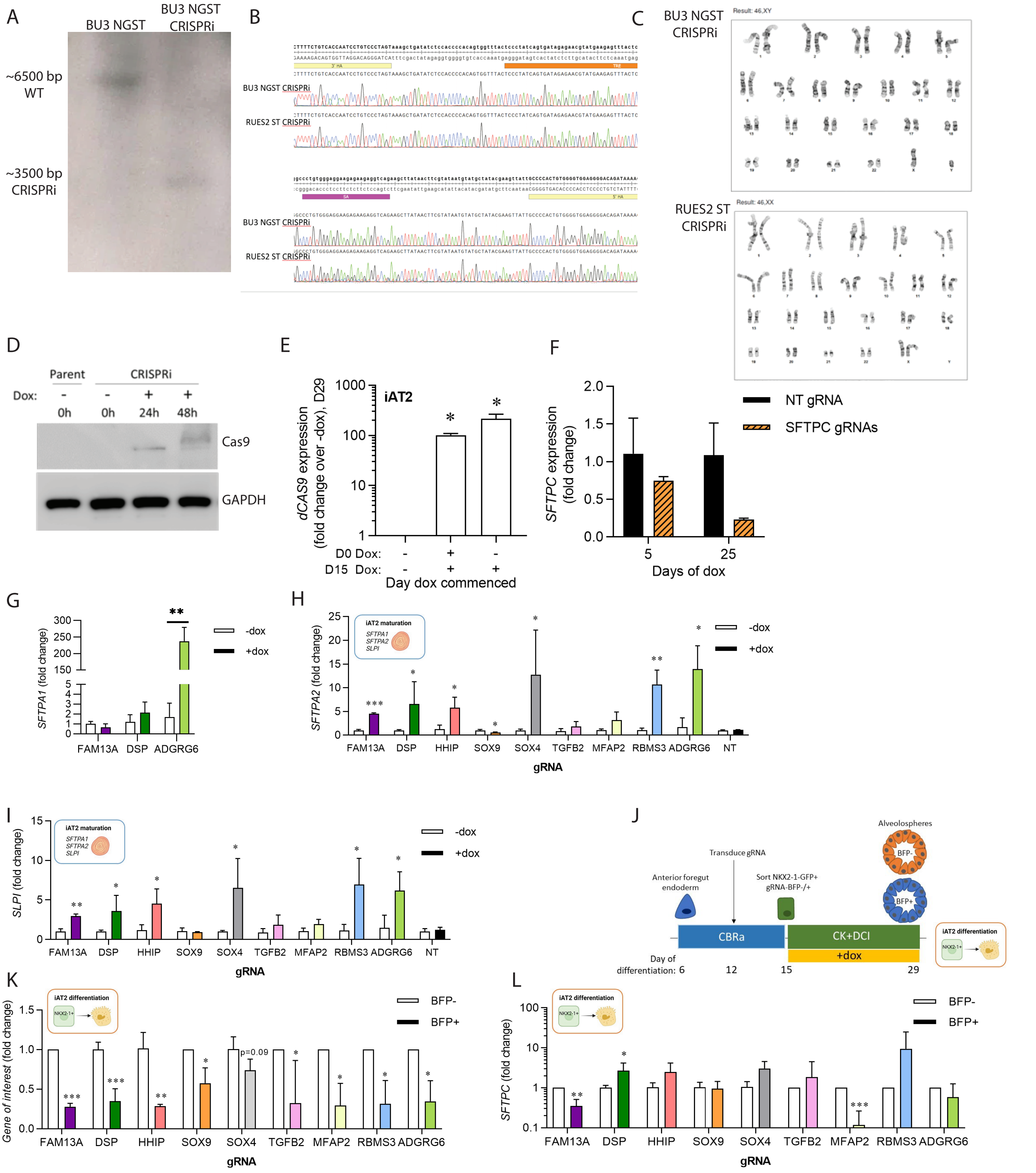

Fig. S1. CRISPRi validation in iPSCs, lung progenitors and iAT2s.

A) Southern blot and B) Sanger Sequencing confirming on-target integration of CRISPRi in the AAVS1 locus.

C) Karyotype of BU3 NGST CRISPRi and RUES2 ST CRISPRi iPSC lines. D) BU3 NGST CRISPRi iPSCs were treated with doxycycline for 24 or 48 hours and Cas9 protein was confirmed by Western blot. E) iPSCs (D0) underwent directed differentiation to lung progenitors (D15) then iAT2s (D29). Cells were treated continuously either with doxycycline from D0, or from D15, then Cas9 mRNA was measured by qRT-PCR at D29. F) iAT2s were transduced with lentiviral-gRNA targeting SFTPC and expression was measured 5 or 25 days following addition of doxycycline. G) RUES2 ST CRISPRi iAT2s SFTPA1 expression measured by qRT-PCR. H) BU3 NGST CRISPRi iAT2s SFTPA2 and I) SLPI expression measured by qRT-PCR. J) BU3 NGST CRISPRi iPSCs underwent directed differentiation to lung progenitors. At day 12 of the differentiation, cells were transduced with lentiviral-gRNA. At day 15 of the differentiation, cells were sorted for NKX2-1-GFP+ lung progenitors and for gRNA-BFP. BFP+ or BFP- cells were replated in CK+DCI supplemented with doxycycline and collected for RNA two weeks later. K) CRISPRi-knockdown was confirmed for each gRNA by qRT-PCR for the gene of interest, relative to BFP- iAT2s, and L) SFTPC expression was measured by qRT-PCR. NT = non-targeting gRNA. n = 3 experimental replicates of independent wells of one differentiation (D-I) or n = 3-5 independent differentiations (K-L, where each individual differentiation BFP- sample is set to 1). All error bars represent SD. Statistical significance was determined using unpaired, two-tailed Student's t test or a one way-ANOVA with a Tukey multiple-comparison test; *p < 0.05, **p < 0.005, and ***p < 0.001.

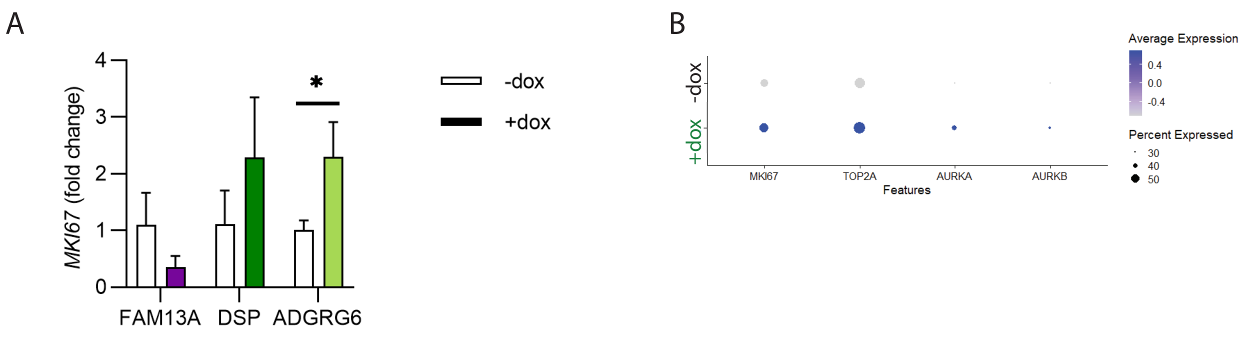

Fig. S2. Effect of CRISPRi knockdown on proliferation.

A) MKI67 expression in RUES2 ST CRISPRi iAT2s. B) Expression of proliferation genes in single-cell RNA-seq data in control (-dox) and DSP-kd (+dox) iAT2s.

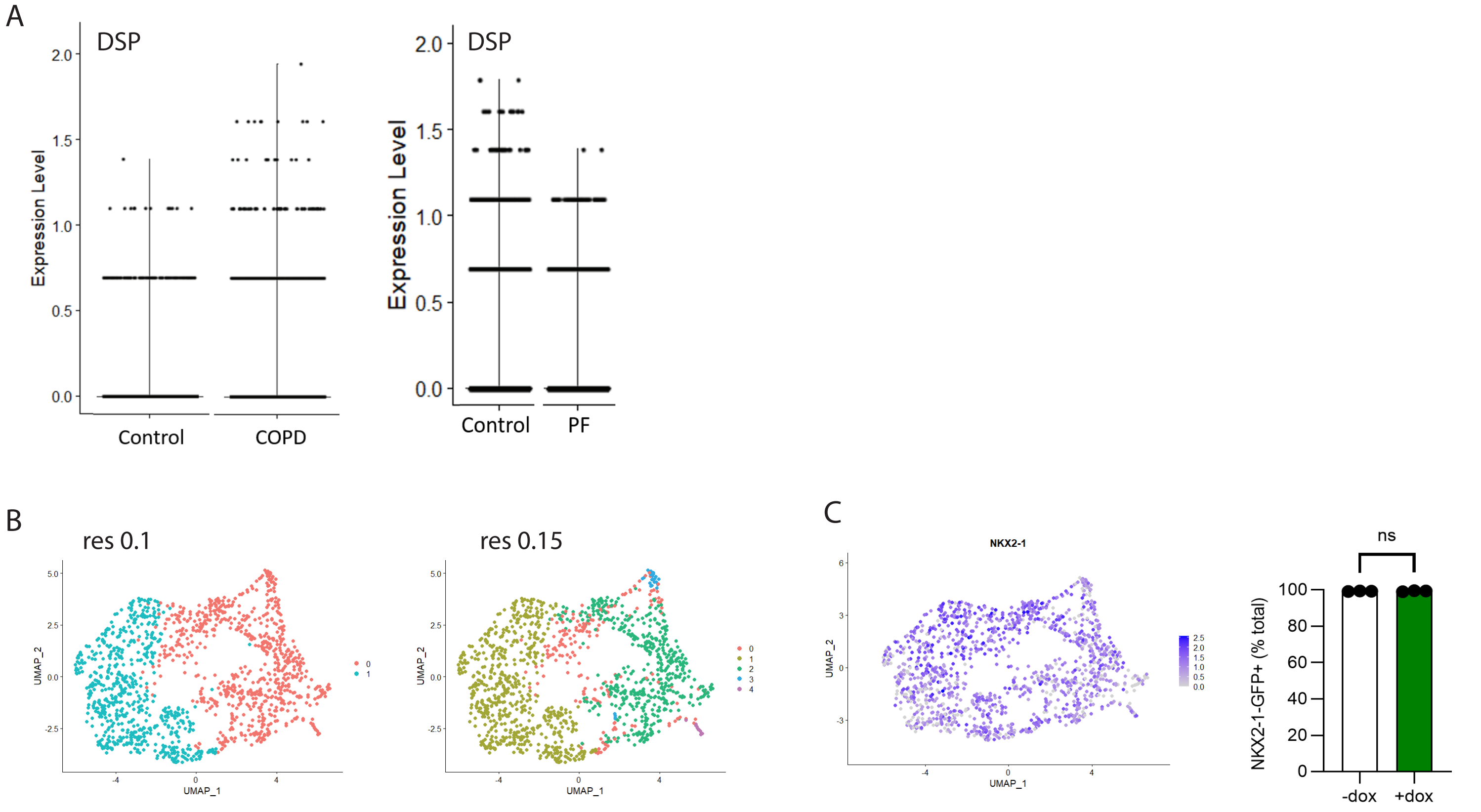

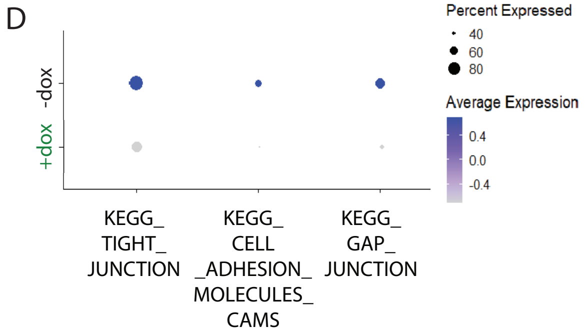

E

	pval	fdr
KEGG_TIGHT_JUNCTION	5.20E-06	0.00097
KEGG_CELL_ADHESION_MOLECULES_CAMS	3.80E-05	0.0035
KEGG_GAP_JUNCTION	0.00012	0.0073

Fig. S3. DSP expression in AT2s, and single-cell RNA-seq of DSP-kd iAT2s.
A) DSP expression in AT2s from control, chronic obstructive pulmonary disease (COPD) or pulmonary fibrosis (PF) human lungs. B) Louvain clustering resolution of 0.1 (left) or 0.15 (right) of single-cell RNA-sequencing data of control (-dox) or DSP-kd (+dox) iAT2s. C) UMAP of NKX2-1 expression (left) or quantified by flow cytometry for the NKX2-1-GFP reporter (right). D) Dot plot of module scores for KEGG Tight Junction, Cell Adhesion Molecules, and Gap Junction. E) Gene-set enrichment analysis of top-differentially expressed genes in control (-dox) iAT2s, relative to DSP-kd (+dox).

A

GSEA – upregulated genes in DSP knockdown

	pval	fdr
KEGG_PPAR_SIGNALING_PATHWAY	2.40E-07	4.50E-05
KEGG_OXIDATIVE_PHOSPHORYLATION	3.20E-05	0.003
KEGG_DRUG_METABOLISM_CYTOCHROME_P450	5.50E-05	0.0034
KEGG_PEROXISOME	8.60E-05	0.0036
KEGG_GLUTATHIONE_METABOLISM	9.80E-05	0.0036

	pval	fdr
HALLMARK_XENOBIOTIC_METABOLISM	3.20E-15	1.60E-13
HALLMARK_ADIPOGENESIS	5.10E-13	1.30E-11
HALLMARK_COAGULATION	5.40E-10	7.70E-09
HALLMARK_OXIDATIVE_PHOSPHORYLATION	6.20E-10	7.70E-09
HALLMARK_FATTY_ACID_METABOLISM	2.90E-08	2.90E-07

B

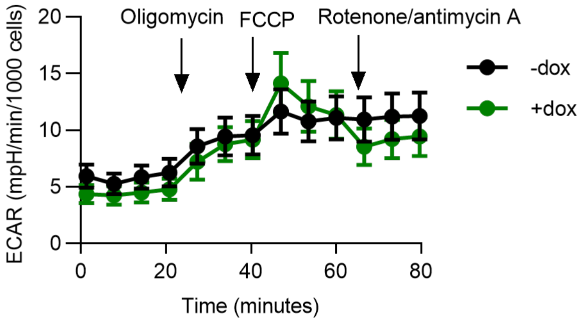

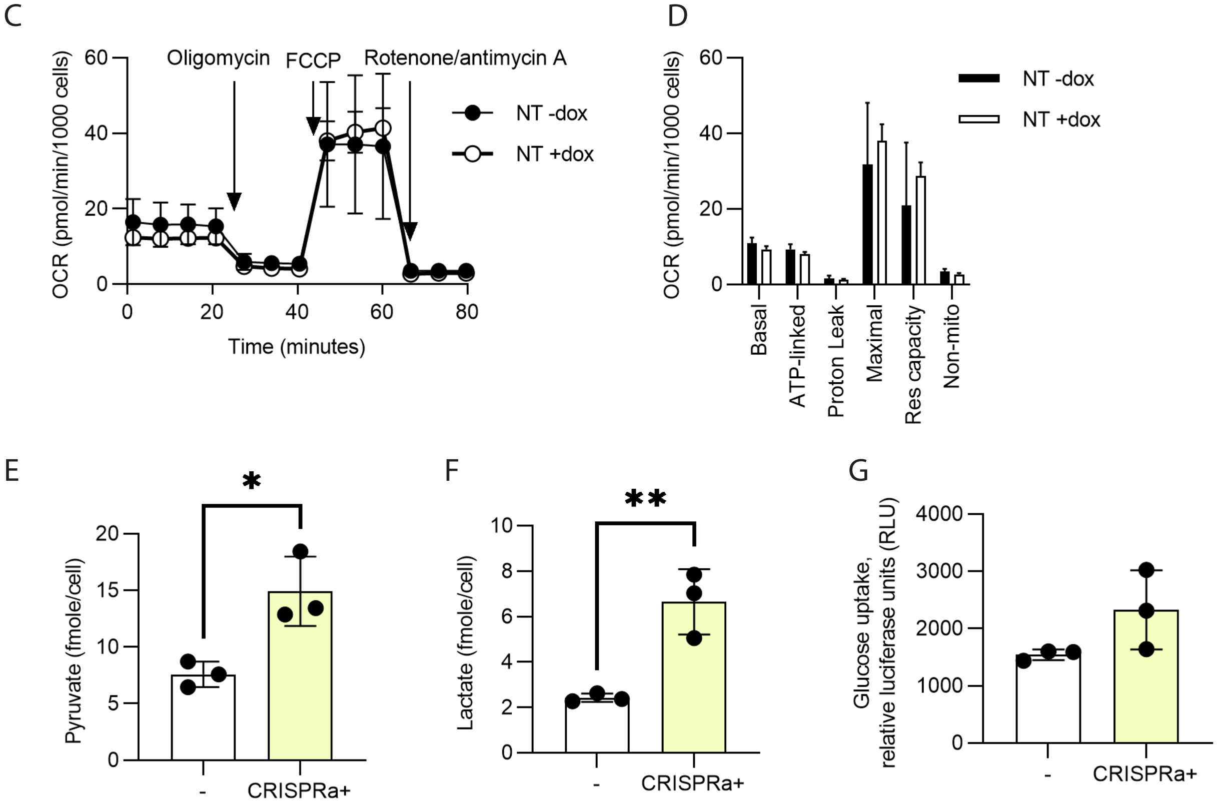

Fig. S4. Effect of DSP expression modulation on mitochondrial respiration.
A) Gene-set enrichment analysis (GSEA) of top-differentially expressed genes in DSP-kd (+dox) iAT2s, relative to control (-dox). B) Extracellular acidification rate (ECAR). Data was normalized by cell count after the assay was complete. n = 6 technical replicates of a differentiation. C-D) Oxygen consumption rate (OCR) was measured in iAT2s transduced with non-targeting (NT) gRNA. Data was normalized by cell count after the assay was complete. n = 6 technical replicates of a differentiation. E) CRISPRa DSP-gRNA iAT2 supernatant was collected and measured for pyruvate and F) lactate. G) Glucose uptake in CRISPRa DSP-gRNA iAT2s. n = 3 experimental replicates of independent wells of one differentiation. All error bars represent SD. Statistical significance was determined using unpaired, two-tailed Student's t-test, *p < 0.05, **p < 0.005, and ***p < 0.001.

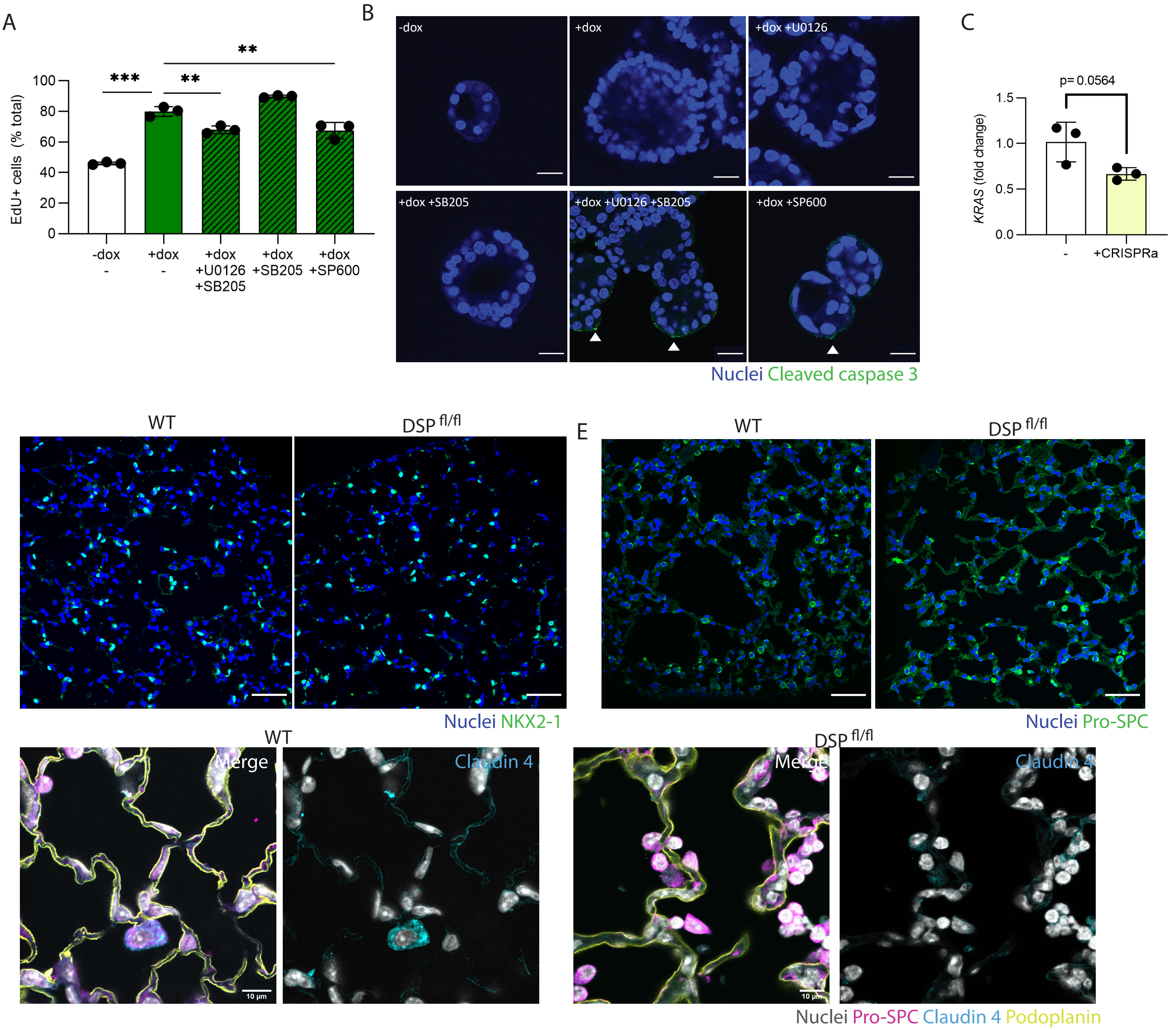

Fig. S5. Expression of DSP regulates ERK signaling, and proliferation in vivo.
A) iAT2s were treated with MAPK inhibitors for 3 days, and pulsed with EdU 24 hours prior to collection. EdU incorporation was measured by flow cytometry. B) iAT2s were treated as in A, then fixed and stained for cleaved caspase 3 (green). Scale bar = 20 uM. C) KRAS expression in iAT2s transduced with CRISPRa-lentivirus and DSP-targeting gRNA-lentivirus was measured by qRT-PCR. D) Nkx2.1-Cre mice were crossed with Dspfl/fl mice and samples collected from uninjured lungs. Stained for NKX2-1 and E) Pro-SPC. Scale bar = 50 uM. F) Nkx2.1-Cre mice were crossed with Dspfl/fl mice. Mice were inoculated with 200 pfu of influenza (IAV) and sacrificed at 10 days post infection. Pro-SPC (magenta), Claudin-4 (cyan), Podoplanin (yellow) and nuclei (grey) were stained for immunofluorescence. Scale bar = 10 uM. n = 3 experimental replicates of independent wells of one differentiation, or 4 mice per group. All error bars represent SD. Statistical significance was determined using unpaired, two-tailed Student's t-test, or a one way-ANOVA with a Tukey multiple-comparison test; *p < 0.05, **p < 0.005, and ***p < 0.001.

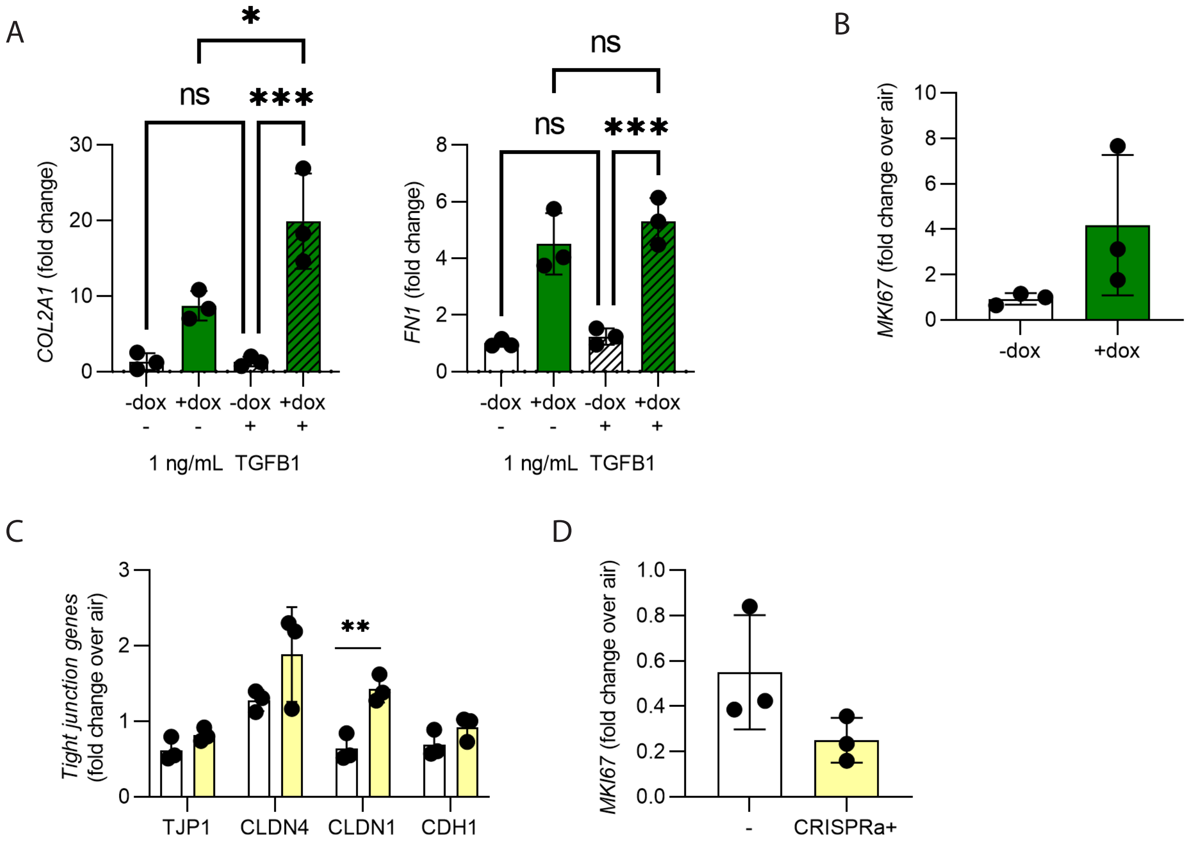

Fig. S6. DSP regulates iAT2 response to injury.
A) Cells were treated with 1 ng/mL TGFβ1 for 48 hours, prior to collecting RNA and COL2A1 and FN1 expression was measured by qRT-PCR. B) CRISPRi DSP-gRNA cells were exposed to cigarette smoke and MKI67 expression measured. C) CRISPRa DSP-gRNA cells were exposed to cigarette smoke and tight junction genes or D) MKI67 expression was measured. n = 3 experimental replicates of independent wells of one differentiation. All error bars represent SD. Statistical significance was determined using unpaired, two-tailed Student's t-test, or a one way-ANOVA with a Tukey multiple-comparison test; $*p < 0.05$, $**p < 0.005$, and $***p < 0.001$.

Table S1. gRNA sequences used in this study

Gene target	Protospacer sequence
NT	GTATTACTGATATTGGTGGG
SFTPC g1	GATGGATGTGGGCAGCAAAG
SFTPC g2	ACCTGCAGCAAGATGGATGT
SFTPC g3	ACGCAACCACACTCACCGGC
FAM13A g1	AGCTGCTCTTCGCTTACCTT
FAM13A g2	CTAGCCATCTGTGTAAGTAT
FAM13A g3	GTCGTTTTAATATTTTCGTC
DSP g1	CGGGTGTCACCGACGCGCTC
DSP g2	CAGAGGAGCTGCGTCGGAGC
DSP g3	ACCCTGGGAAGAAACCGGCC
DSP RNP gRNA	AGGATGTACTATTCTCGGCG
MFAP2 g1	ACTTACCAGGCAGGAATAGC
MFAP2 g2	GGGGGTAGCTCCTCTTATGT
MFAP2 g3	CTGAGGAGTAGGGTTAGTAG
MFAP2 g4	TTGTCACGACTTATGACCAG
RBMS3 g1	CCCCGAACACCATGTCACTC
RBMS3 g2	AGCAGCAACTAAGCTGTACA
RBMS3 g3	GAAGCTGATCTGCAAGGATT
RBMS3 g4	TTACGAACGCTGGCAATTGA
SOX4 g1	AAGAGGCCTGTTTCGCTGTC
SOX4 g2	GTTTCGCTGTCGGGTCTCTA
SOX4 g3	ACTCCTTAGTGCCGATTCCG
SOX4 g4	GAGCTACCGAGAGCGCTCGT
SOX9 g1	ATTCGCCTCCCCCCACTTGG
SOX9 g2	TAAGTGCTCGCCGCGGTAGC
SOX9 g3	GGAGCCGCTTGCTCCGCATC
SOX9 g4	CTCGAGTCCCCGAGCCGCCG
TGFB2 g1	CCCCGGCAAGATCGTGATGT
TGFB2 g2	AGCAGAAGGTTCGCTCCGAG
TGFB2 g3	AATATTAGCCTGACGGTCTA
TGFB2 g4	CTAAGCGAGCAATTCCACGT
HHIP g1	CTTCTTTTTAACTAGCGCGC
HHIP g2	GCGAGAAGCGGTGACGTCAA
HHIP g3	TGTTCACTTGTCCGTGTAAC
HHIP g4	AGGAACAGAAACGGCGACGG
ADGRG6 g1	CAGGGTTCACCTTGCGCCGT
ADGRG6 g2	ACCCTCCTTTACGCCGTTTC
ADGRG6 g3	GAGGATGATCTTGCGGCCAA
ADGRG6 g4	TACTGCGCCGAGGTCCCCTT
